# An siRNA-guided ARGONAUTE protein directs RNA polymerase V to initiate DNA methylation

**DOI:** 10.1038/s41477-021-01008-7

**Published:** 2021-11-08

**Authors:** Meredith J. Sigman, Kaushik Panda, Rachel Kirchner, Lauren L. McLain, Hayden Payne, John Reddy Peasari, Aman Y. Husbands, R. Keith Slotkin, Andrea D. McCue

**Affiliations:** 1 Department of Molecular Genetics, The Ohio State University, Columbus, OH USA; 2 Donald Danforth Plant Science Center, St. Louis, MO USA; 3 Bioinformatics and Computational Biology Program, Saint Louis University, St. Louis, MO USA; 4 Division of Biological Sciences, University of Missouri, Columbia, MO USA; 5 Present Address: Medical Scientist Training Program, University of Wisconsin, Madison, WI USA; 6 Present Address: Graduate Program in the School of Plant Sciences, University of Arizona, Tucson, AZ USA

**Keywords:** Transgenic plants, DNA methylation, RNAi

## Abstract

In mammals and plants, cytosine DNA methylation is essential for the epigenetic repression of transposable elements and foreign DNA. In plants, DNA methylation is guided by small interfering RNAs (siRNAs) in a self-reinforcing cycle termed RNA-directed DNA methylation (RdDM). RdDM requires the specialized RNA polymerase V (Pol V), and the key unanswered question is how Pol V is first recruited to new target sites without pre-existing DNA methylation. We find that Pol V follows and is dependent on the recruitment of an AGO4-clade ARGONAUTE protein, and any siRNA can guide the ARGONAUTE protein to the new target locus independent of pre-existing DNA methylation. These findings reject long-standing models of RdDM initiation and instead demonstrate that siRNA-guided ARGONAUTE targeting is necessary, sufficient and first to target Pol V recruitment and trigger the cycle of RdDM at a transcribed target locus, thereby establishing epigenetic silencing.

## Abstract (web summary)

This study finds that siRNA-guided ARGONAUTE first recruits polymerase V to new target sites without pre-existing DNA methylation and triggers the cycle of RdDM at the target sites, thereby establishing epigenetic silencing.

## Main

Chromatin marks segregate genomes into expressed domains and regions that remain transcriptionally silenced. In mammals and plants, DNA methylation provides information such as which regions are transposable elements (TEs) and should not be expressed (reviewed in ref. ^1^), while integrated transgenes are often subject to this regulation as well^2^. Most studies have focused on how DNA methylation is epigenetically maintained, resulting in heritable transcriptional repression. However, how DNA methylation is initially established at individual loci is less understood.

In both plants and mammals, DNA methylation is targeted via the action of small RNAs (piRNAs in mammals)^3,4^. Specifically in plants, siRNAs are produced from TEs, viruses and transgenes, targeting them for RdDM (reviewed in ref. ^5^). RdDM is a feed-forward cycle that reinforces DNA methylation and results in epigenetic transcriptional repression. The mechanism of RdDM is split between an upstream siRNA-generating phase and a downstream chromatin-linked phase. In the upstream phase, siRNAs are generated from either RNA polymerase IV (Pol IV)- or Pol II-derived transcripts. In the downstream phase, these siRNAs are incorporated into one of the closely related ARGONAUTE proteins AGO4, AGO6 or AGO9 (ref. ^6^). Base complementarity between the siRNA and a chromatin-linked nascent transcript results in recruitment of the de novo DNA methyltransferases DRM1 and DRM2 (ref. ^7^). The nascent transcript, produced by RNA polymerase V (Pol V), provides the scaffold for AGO–siRNA complex interaction^8,9^. Pol IV and Pol V are derived from Pol II, as subunits of these holoenzymes duplicated early in plant evolution and subfunctionalized into their respective roles in siRNA biogenesis (Pol IV) and scaffolding RNA production (Pol V)^10^.

Pol V is continually recruited to RdDM loci through its interaction with the proteins SUVH2 and SUVH9, which bind existing cytosine DNA methylation^11,12^. However, how Pol V is recruited to new unmethylated DNA to first trigger RdDM and establish chromatin marks is not understood. One popular model in the literature proposes that, in the absence of Pol V, a Pol II-derived transcript acts as the scaffold and can set the initial round of DNA methylation^13,14^. A second model suggests that Pol V ubiquitously surveys the genome at low levels, and the first round of RdDM occurs upon the addition of siRNAs. This ‘Pol V surveillance’ model is supported by a recent publication demonstrating that Pol V is present at a second set of loci that do not undergo RdDM due to lack of siRNAs^15^. In both models, after the first round of methylation, Pol V would then be recruited through the activity of SUVH2/SUVH9, leading to the positive feed-forward loop of RdDM. Neither of the two models have been examined in the context of true ‘de novo’ silencing as in ref. ^16^, and this leaves a formidable gap in our understanding of how the first round of DNA methylation is established.

Since RdDM is a self-reinforcing cycle, it is impossible to study the first round for endogenous regions of the genome that are already engaged. To address this, we developed an approach that interrogates de novo DNA methylation of newly transformed transgene DNA, so that no pre-existing chromatin marks are guiding RdDM. We used this system to address a critical question in plant epigenetics: how is Pol V first recruited to new unmethylated sites for the first round of RdDM? Our findings demonstrate that, contrary to previous models, AGO4-clade proteins precede Pol V recruitment to new targets of RdDM. This mechanism provides the missing link between unmethylated DNA and the initiation of chromatin modification towards epigenetic silencing.

## Results

### Transgenic system to investigate the first round of DNA methylation

**Fig. 1:**
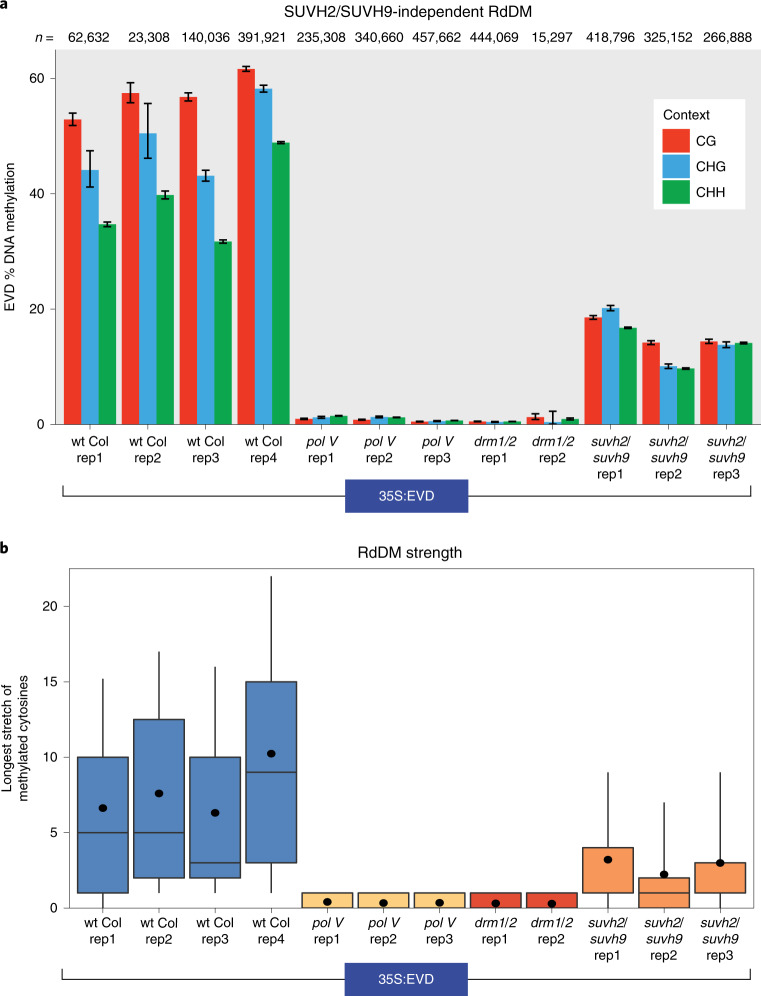
SUVH2/SUVH9-independent function of Pol V. **a**, BSAS of the 35S:EVD transgene in independent biological replicates of T1 plants. The bar displays the methylation percentage and the error bars represent the 95% confidence interval calculated using the Wilson score interval method. *n* is the number of total cytosines assayed for each amplicon. H is A, C or T. **b**, RdDM strength plot of DNA methylation. RdDM strength was measured by calculating the length of stretches of consecutively methylated cytosines along individual sequence reads. All box plots in this paper represent 25th and 75th percentile values with whiskers at the 10th and 90th percentiles, the median is represented by a line and the mean is denoted by a filled circle.

The expression of TE-derived sequences reproducibly triggers RdDM^17,18^. To study the initiation of DNA methylation, we recreated the ‘35S:EVD’ transgene consisting of a broadly expressed promoter driving the full-length coding sequence of the *Arabidopsis* *Evadé* TE^19^. This transgene was stably integrated into wild-type Columbia (wt Col) *Arabidopsis thaliana* genomes, and transgene silencing in the first-generation (T1) transformants was assayed for all experiments. To determine whether our TE transgene triggers RdDM, we used bisulfite amplicon sequencing (BSAS)^20^, a technique with high sequencing depth. In a side-by-side comparison, the average methylation remains the same for BSAS, Sanger sequencing and whole-genome sequencing techniques at target loci after conversion with sodium bisulfite, but the resolution of the data with BSAS is superior (roughly 3,800 average coverage) (Supplementary Fig. 1). When BSAS and small RNA sequencing were applied to T1 35:EVD plants, we found that siRNAs are generated from the transgene (Supplementary Fig. 2), which results in high levels of de novo DNA methylation (Fig. 1a and Supplementary Fig. 2). Furthermore, although the coding sequence in 35S:EVD is an exact copy of the *Evadé* element EVD5 from the *Arabidopsis* genome (At5TE20395), the transgene is not competent to transpose (Supplementary Fig. 2), and transgene methylation is not influenced in *trans* by siRNAs from the endogenous EVD elements (Supplementary Fig. 2).

### SUVH2/SUVH9-independent Pol V function

Pol II has been proposed to substitute for Pol V during the first round of RdDM. To test this, we interrogated the methylation of T1 35S:EVD in *pol V* mutants (*nrpE1* subunit mutation). We found that Pol V is necessary to trigger RdDM, as plants lacking Pol V have only background levels of methylation similar to plants lacking de novo DNA methyltransferase activity (in the *drm1/2* double mutant), and similar to the non-conversion rate during sodium bisulfite treatment (Fig. 1a) (non-conversion rates calculated in Supplementary Fig. 1). BSAS affords increased data resolution^20^, which reveals that the low level of DNA methylation in *pol V* mutants exists as sporadic unconverted cytosines (stochastic), rather than as consecutive runs of methylated cytosines (indicative of DRM1/DRM2 activity in RdDM) (Supplementary Fig. 1). We assayed RdDM strength by quantifying these strings of consecutive methylated bases (Methods and controls in Supplementary Fig. 1), which takes advantage of the large number of reads generated by BSAS to individually score each read, rather than averaging the data as in other methylation analysis methods. Using this improved methodology, we found no evidence of RdDM activity in *pol V* mutants (Fig. 1b). Our data demonstrate that, contrary to the Pol II substitution model, Pol V is essential to initiate the first round of DNA methylation.

The only known mechanism for directly recruiting Pol V is through the DNA methyl-binding proteins SUVH2/SUVH9 (refs. ^11,12^). We found that the DNA methylation of 35S:EVD in plants lacking both SUVH2 and SUVH9 does not phenocopy the total loss of RdDM in *pol V* or *drm1/2* mutants (Fig. 1a). Rather, *suvh2/suvh9* double mutants have an intermediate level of DNA methylation and RdDM strength (Fig. 1a,b), suggesting that these proteins function to amplify DNA methylation levels rather than trigger RdDM at new locations. This demonstrates that there is a mechanism other than SUVH2/SUVH9 for the first round function of Pol V at an unmethylated target locus.

### AGO4-clade proteins are required for the first round of RdDM

**Fig. 2:**
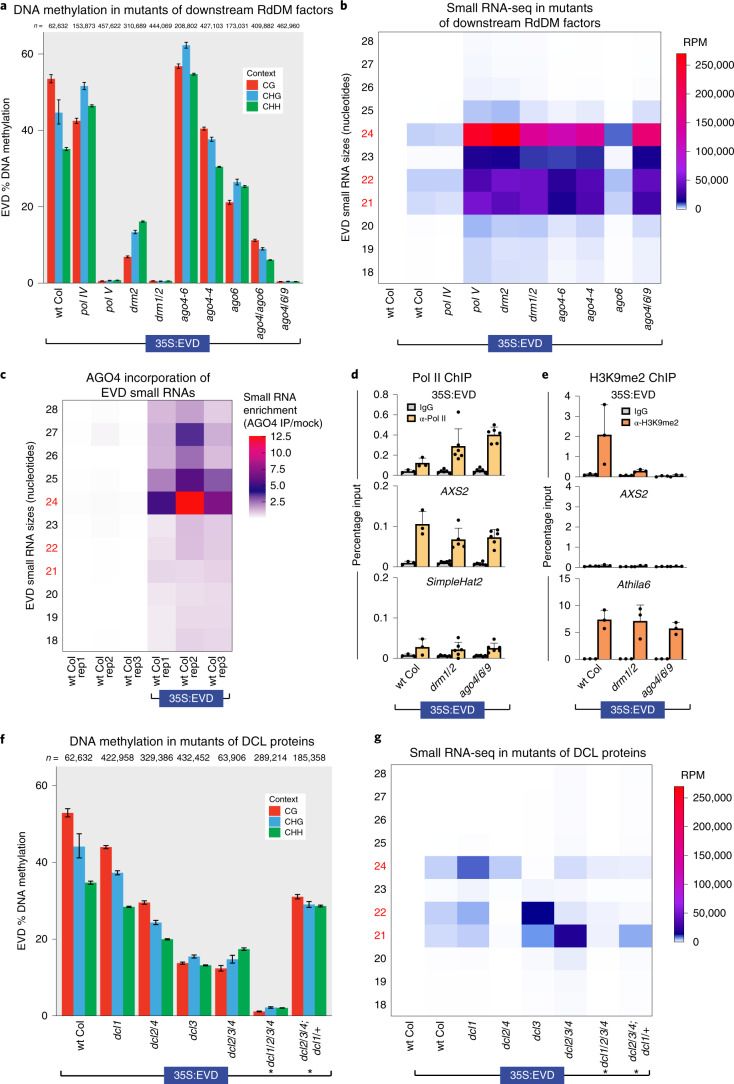
Genetic requirements for the first round of RdDM. **a**, BSAS of 35S:EVD in T1 plants lacking key RdDM factors demonstrates that the initiation of DNA methylation is dependent on Pol V, DRM1/2 and at least one member of the AGO4-clade of proteins. The data display is the same as Fig. 1a. The wt Col and *pol V* data are replicates 1 and 3, respectively, from Fig. 1a. **b**, Heatmap of small RNA sequencing for transgenic lines from **a**. The accumulation of differently sized siRNAs is shown on the *y* axis, with the key sizes 21, 22 and 24 nt in red. The siRNAs shown were mapped to the entire length of the EVD coding region, not just the region analysed in **a** with BSAS. **c**, Heatmap of EVD small RNA enrichment in AGO4. Enrichment is calculated as the ratio of small RNA accumulation (RPM) in AGO4-IP over mock-IP samples for each size class of small RNA. **d**, ChIP analysis of Pol II (Ser5P) in T1 transgenic plants from **a** shown as percentage input. Error bars represent the standard deviation of the mean. Three biological replicates were used for wt Col, and six biological replicates were used for the other two genotypes. *SimpleHat2* is a transcriptionally silenced TE negative control, and *AXS2* is a transcribed positive control gene. **e**, ChIP analysis of H3K9me2 levels in T1 transgenic plants shown as percentage input. Three biological replicates were used for each genotype. *Athila6* is a silenced TE with known accumulation of H3K9me2. Error bars represent the standard deviation of the mean of three biological replicates. **f**, BSAS of 35S:EVD in T1 plants lacking various combinations of DCL family proteins. The *dcl1/2/3/4* quadruple mutant plants are siblings of the *dcl2/3/4*; *dcl1*/+ plants (siblings are denoted with an asterisk). The data display is the same as Fig. 1a. The wt Col data are replicate 1 from Fig. 1a. **g**, Heatmap of small RNA sequencing for the same transgenic lines as in **f**. The wt Col data are the same from **b**.

To determine which other pathway components are essential for the first round of RdDM, we transformed 35S:EVD into a series of known DNA methylation, RdDM and RNA interference (RNAi) mutants. As expected, 35S:EVD methylation and siRNA production are not dependent on DNA methyltransferases that propagate DNA methylation during DNA replication (maintenance methyltransferases) (Supplementary Fig. 3). Rather, its methylation is dependent on at least one of the closely related AGO4-clade proteins (AGO4, AGO6 or AGO9 in the *ago4/6/9* triple mutant) (Fig. 2a, Supplementary Fig. 4). Because AGO2 has a published role in RdDM^21^, we additionally tested *ago2* mutants and found only partial reductions in 35S:EVD 21–22-nucleotide (nt) siRNAs, DNA methylation and RdDM strength, suggesting it plays only a secondary role during the initiation of DNA methylation (Supplementary Fig. 5). Furthermore, we used IP followed by small RNA sequencing and found that 35S:EVD-derived siRNAs are enriched in AGO4 protein complexes (Fig. 2c and controls in Supplementary Fig. 6), demonstrating the direct role of AGO4 in the RdDM of 35S:EVD.

SiRNA biogenesis is not dependent on Pol V, DRM1/DRM2 or AGO4/AGO6/AGO9 (Fig. 2b and Supplementary Fig. 4), confirming that these proteins function downstream of siRNA production during the chromatin-linked phase of RdDM. Instead, mutations in the downstream factors Pol V, DRM1/DRM2 and AGO4 have increased siRNA accumulation (Fig. 2b). We found that this increase in siRNAs positively correlates with the level of Pol II transcription of 35S:EVD in *drm1/2* and *ago4/6/9* mutants (Fig. 2d), and is inversely correlated with the level of the transcriptionally repressive histone mark H3K9me2 (Fig. 2e). This demonstrates that in mutants of RdDM downstream machinery (*pol V*, *drm1/2*, *ago4/6/9*), without DNA methylation and H3K9me2, 35S:EVD expression is uninhibited, generating more transcription that leads to increased siRNA production. Additional regions of the endogenous genome also generate more siRNAs in *pol V*, *drm1/2* and *ago4/6/9* mutants (Supplementary Fig. 7), suggesting that a lack of downstream RdDM function broadly leads to enhanced Pol II transcription and increased siRNA production.

### Pol II-derived transcripts cleaved by DICER proteins trigger RdDM

Our data indicate that siRNA production from 35S:EVD is from Pol II, since siRNA accumulation is not lost in *pol IV* or *pol V* mutants (Fig. 2b), and the abundance of siRNAs (Fig. 2b) positively correlates with the level of Pol II transcription of 35S:EVD (Fig. 2d). In plants, siRNA sizes and categories are determined by the specific DCL protein that produces them^22^. Our data refute an existing ‘saturation’ model based on the identical 35S:EVD transgene, in which RdDM begins only when DCL2 and DCL4 are overwhelmed with double-stranded RNA substrate, thus activating DCL3 to generate 24-nt siRNAs^19^. Instead, we find that *dcl2/4* double mutants result in a roughly 50% reduction in DNA methylation (Fig. 2f) rather than being hyper-methylated as previously posited^19^. Further, DNA methylation is not entirely dependent on the presence of 24-nt siRNAs, as *dcl3* mutants that lack 24-nt siRNAs (Fig. 2g) do not lose all methylation (Fig. 2f) (as in ref. ^16^). Additionally, DNA methylation still persists in *dcl2/3/4* triple mutants, where only DCL1-dependent 21-nt siRNAs remain (Fig. 2f,g). These data indicate that 21, 22 and 24-nt siRNAs are all sufficient to trigger the initiation of RdDM. DNA methylation is nearly absent only when siRNAs are severely reduced in *dcl1/2/3/4* quadruple mutants (Fig. 2f,g). Therefore, the initiation of DNA methylation is fully dependent on the presence of Pol II-derived DCL-processed siRNAs in a size-independent manner.

### Initial Pol V localization requires an AGO4-clade protein

**Fig. 3:**
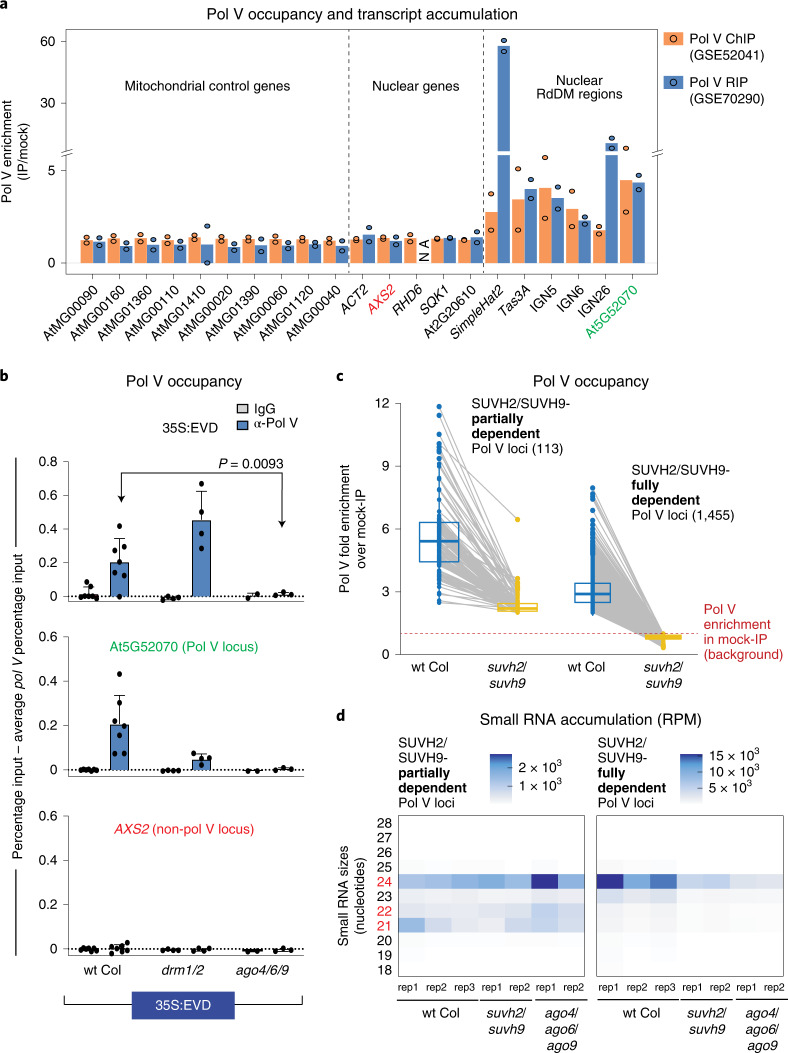
The first round of Pol V recruitment requires an AGO4-clade protein. **a**, Reanalysis of ChIP and RIP data of Pol V occupancy and transcription. Mitochondrial negative control genes, nuclear genes and nuclear positive control RdDM regions are shown. Two replicates are shown as dots, and their average is the height of the bar. NA, not applicable due to absence of any mapping reads in the IP or the mock samples. Genes with coloured labels are used as controls in ChIP experiments. **b**, Pol V ChIP of 35S:EVD T1 plants as percentage input minus the background of percentage input in T1 *pol V* mutants. At least three biological replicates were used for each genotype: seven for wt Col, four for *drm1/2* and three for *ago4/6/9*. At5G52070 is a locus that undergoes RdDM and serves as a positive control for Pol V occupancy^32^, while *AXS2* is a gene that does not undergo RdDM and serves as a negative control. Error bars represent the standard deviation of the mean. Statistical significance was determined by a two-tailed unpaired *t*-test with Welch’s correction. **c**, Box plot with connected data points of Pol V occupancy comparing wt Col (blue) and *suvh2/suvh9* double mutants (yellow). Pol V occupied regions are categorized into SUVH2/SUVH9-partially and -fully dependent loci (Methods). Pol V occupancy is displayed as fold enrichment over mock-IP. Grey lines represent the change in Pol V enrichment between wt Col and *suvh2/suvh9* for each locus. Box plots represent 25th and 75th percentile with whiskers at 10th and 90th percentile values, and the median is represented by a line. **d**, Heatmap of small RNA sequencing for Pol V occupied loci that are categorized as either SUVH2/SUVH9-partially or -fully dependent from **c**.

As Pol V is essential to establish RdDM, (Fig. 1), we therefore aimed to understand how Pol V is recruited to a locus in the absence of pre-existing DNA methylation. We reanalysed published Pol V chromatin immunoprecipitation (ChIP) and RNA IP (RIP) data^9,12^ to determine whether Pol V is present (ChIP) and transcribing (RIP) at low levels throughout the genome. Our reanalysis centred on using mitochondrial genes as additional negative baseline controls, as Pol V subunits are located exclusively in the nucleus^23,24^. We find that Pol V signal is detected at non-RdDM nuclear loci only at the same rate as the mitochondrial negative control genes (Fig. 3a). Therefore, rather than patrolling all loci at low levels, Pol V is actively recruited to its new target sites for the initiation of RdDM.

We identified three mutant combinations of downstream RdDM factors that completely lose the ability to target T1 35S:EVD for RdDM (*pol V*, *drm1/2* and *ago4/6/9*) (Fig. 2). To test the requirements of Pol V recruitment to new loci, we performed Pol V (subunit NRPE1) ChIP in these mutant backgrounds in the T1 generation of 35S:EVD plants. We found that the Pol V protein still accumulates in each of these mutant backgrounds (Supplementary Fig. 7) and is recruited in the first generation to the TE transgene (Fig. 3b). This recruitment is not dependent on existing DNA methylation, as Pol V recruitment still occurs in the *drm1/2* mutant (Fig. 3b) that lacks DNA methylation (Fig. 1). Therefore, this system provides the ability to dissect the methylation-independent recruitment of Pol V. We found that Pol V is not recruited for the first round of RdDM in the *ago4/6/9* triple mutant (Fig. 3b). This was surprising, as the prevailing dogma suggests that Pol V presence and transcription occurs first and is required to position AGO4-clade proteins at the chromatin target^25^. Conversely, our data demonstrate that AGO4-clade proteins are required to localize Pol V during the initiation of RdDM.

AGO4-clade proteins are directed to their targets by the complementarity of their incorporated siRNAs^26^. To determine whether there are regions of the endogenous genome where Pol V is positioned independently of SUVH2/SUVH9 and instead on the basis of AGO4/siRNAs, we first identified regions of the genome where Pol V occupancy is only partially dependent on the methylation-dependent SUVH2/SUVH9 mechanism (left, Fig. 3c). These 113 regions retain siRNA accumulation in *suvh2/suvh9* and *ago4/6/9* mutants (Fig. 3d). Conversely, the 1,455 regions of the genome where Pol V recruitment is fully dependent on SUVH2/SUVH9 tend to have reduced siRNA accumulation in *suvh2/suvh9* mutants (Fig. 3d). These data indicate that when the SUVH2/SUVH9 recruitment method is absent, the continual production of siRNAs is necessary for Pol V occupancy at a small number of loci in the *Arabidopsis* genome.

### AGO4 localizes independently of Pol V and DNA methylation

**Fig. 4:**
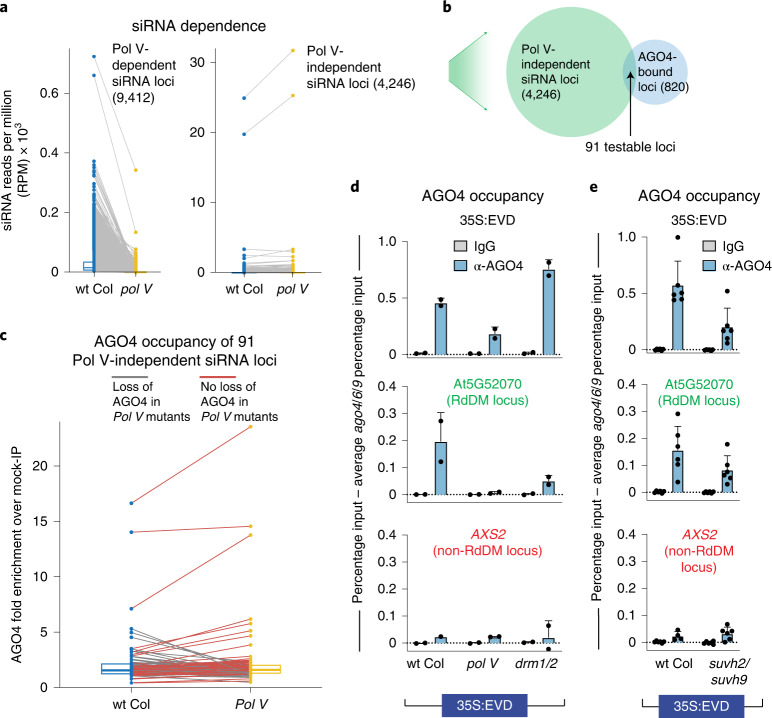
AGO4 can localize to chromatin loci independent of Pol V. **a**, Box plot with connected data points of 23–24-nt siRNA accumulation in wt Col (blue) and *pol V* mutants (yellow). The siRNA loci are defined as Pol V-dependent or -independent on the basis of the change in siRNA accumulation in *pol V* compared to wt Col. Box plot percentiles are the same as in Fig. 3c. **b**, Venn diagram showing the overlap of Pol V-independent siRNA loci from **a** and AGO4-enriched loci as previously defined^27^. The overlap provides 91 testable loci for **c**. **c**, Box plot with connected data points of AGO4 protein enrichment. AGO4 occupancy is displayed as fold enrichment over mock-IP. Grey lines display loci that lose AGO4 occupancy in *pol V* mutants (31%). Red lines display loci that retain AGO4 occupancy in the *pol V* mutant background (69%). Box plot display is the same as in Fig. 3c. **d**, AGO4 ChIP of 35S:EVD T1 plants as percentage input minus the background of percentage input in T1 *ago4/6/9* mutants. Bar graph display is the same as in Fig. 3b. At5G52070 is a positive control locus that undergoes RdDM, and *AXS2* is a negative control gene that does not go through RdDM. Error bars represent the standard deviation of the mean of two biological replicates. This experiment was performed twice with similar results each time. **e**, AGO4 ChIP of 35S:EVD T1 plants as percentage input minus the background of percentage input in T1 *ago4/6/9* mutant plants. Bar graph display is the same as in Fig. 4d. Error bars represent the standard deviation of the mean of six biological replicates.

We aimed to order the events of recruitment at the chromatin that result in the first round of RdDM. Since Pol V recruitment to 35S:EVD is dependent on a protein of the AGO4-clade (Fig. 3), we aimed to determine whether the converse was true: is AGO4’s interaction with chromatin dependent on Pol V? Our data support this idea that there are a small number of regions of the endogenous *Arabidopsis* genome where AGO4 interacts with target chromatin loci independent of a Pol V-derived scaffolding transcript. Since AGO4 targeting is dependent on siRNA complementarity, we began by identifying loci that continue to produce 23–24-nt siRNAs in a *pol V* mutant (Fig. 4a). We overlapped these 4,246 Pol V-independent siRNA loci with 820 previously identified AGO4-bound loci^27^, resulting in 91 testable AGO4-bound regions of the genome that do not lose siRNAs (Fig. 4b). Of these testable AGO4-bound regions, 63 (69%) retain AGO4 occupancy in the *pol V* mutant (Fig. 4c), demonstrating AGO4 recruitment without Pol V.

To determine whether AGO4 is localized to the 35S:EVD transgene during the initiation of RdDM, we performed AGO4 ChIP on T1 35S:EVD plants. As a control, we confirmed that the AGO4 protein is present in the various mutants we tested (Supplementary Fig. 7). In the ChIP experiment, we detected AGO4 at 35S:EVD during the initiation of RdDM in wild-type plants, as well as in *pol V*, *suvh2/suvh9* and *drm1/2* mutants (Fig. 4d,e). The level of AGO4 at 35S:EVD in *pol V* and *suvh2/suvh9* mutants is not as high as in wt Col plants, but is nonetheless substantially higher than the negative control *AXS2* gene and the IgG negative control (Fig. 4d,e). In addition, the AGO4 ChIP signal in the *drm1/2* mutant may be higher due to the increased siRNAs produced in this mutant (Fig. 2b). We conclude that AGO4 is directed to the target transgene without the requirement of a Pol V scaffolding transcript or DNA methylation, and is independent of the known SUVH2/SUVH9 recruitment mechanism of Pol V (Fig. 4e), placing AGO4 interaction with the target locus before and not reliant on Pol V activity.

### siRNAs are sufficient to direct Pol V function

**Fig. 5:**
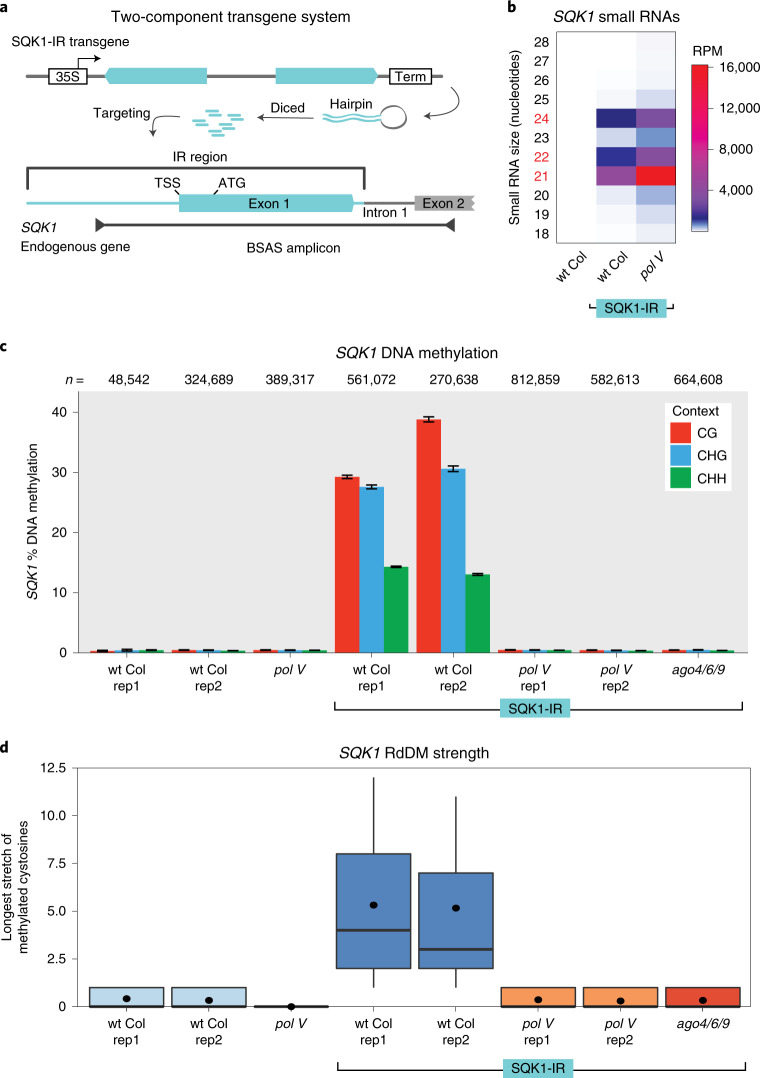
siRNAs are sufficient to target the first round of RdDM. **a**, Two-component system with siRNA production from a T1 inverted repeat (IR) transgene targeting the endogenous gene *SQK1* for RdDM. **b**, Heatmap of small RNA sequencing. **c**, BSAS for T1 transgenic SQK1-IR plants. The data display is the same as Fig. 1a. **d**, Box plots of RdDM strength for plants from **c**. Box plot percentiles are the same as in Fig. 1b.

Since AGO4 can be recruited to a new target locus independent of Pol V, we aimed to determine whether siRNAs are sufficient to direct Pol V activity and the first round of RdDM. Our 35S:EVD transgene system cannot address this question, since the source locus of the siRNAs and the target of Pol V action are the same (*cis*-acting). Instead, we generated a *trans*-acting two-component system to uncouple siRNA production from Pol V recruitment. The siRNAs are generated from an inverted repeat transgene that targets an unmethylated endogenous gene in *trans* (Fig. 5a) (as in refs. ^28–30^). The endogenous gene we targeted (At3G12210) encodes a broadly expressed DNA-binding protein that does not produce siRNAs (Fig. 5b), is not methylated (Fig. 5c) and is not bound by Pol V (Fig. 3a) in wt Col plants. We named this uncharacterized gene *SQUEAKY1* (*SQK1*). On addition of the *SQK1* inverted repeat transgene (SQK1-IR), 21, 22 and 24-nt siRNAs accumulate from the hairpin region (Fig. 5b) and these siRNAs function in *trans* to direct RdDM to the *SQK1* endogenous gene (Fig. 5c,d). In a *pol V* mutant, siRNAs are still generated from the inverted repeat transgene (Fig. 5b), but RdDM does not occur (Fig. 5c,d), again demonstrating that Pol V acts downstream of siRNA production and is essential for RdDM. As with 35S:EVD, the initiation of RdDM is fully dependent on an AGO4-clade protein (Fig. 5c,d). Taken together, these data demonstrate that the production of new siRNAs and the presence of an AGO4-clade protein are sufficient to target Pol V-dependent RdDM to a new non-transgenic locus independent of existing methylation.

### Pol II expression is necessary for the first round of RdDM

**Fig. 6:**
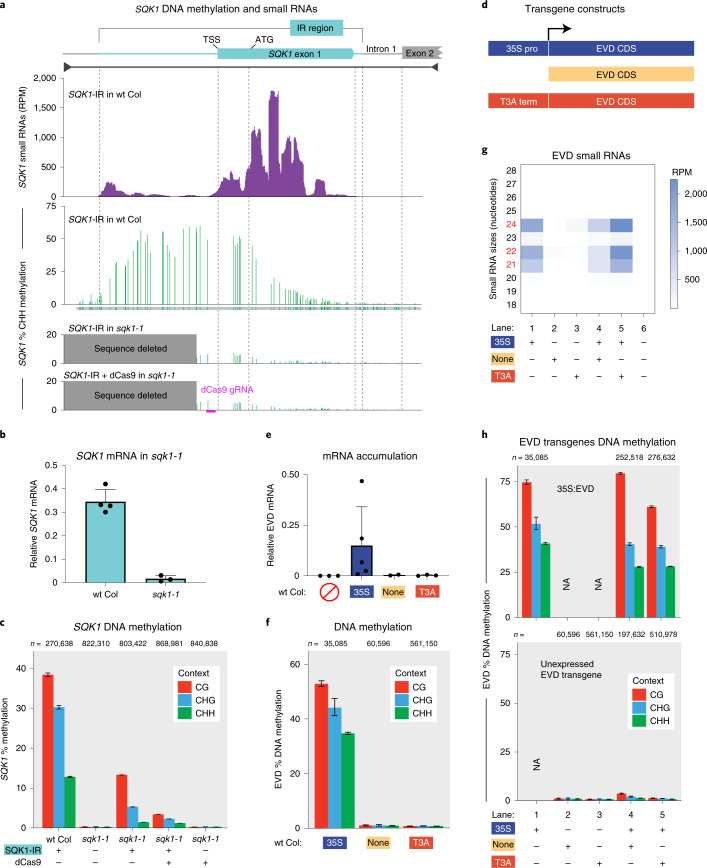
The role of Pol II in the first round of RdDM. **a**, CHH context DNA methylation and siRNAs (combined 21, 22 and 24 nt) aligned to the endogenous *SQK1* gene. The region of *SQK1* that matches the SQK1-IR transgene is annotated. The track below the SQK1-IR of wt Col CHH methylation identifies where each CHH context cytosine is located (green), even if unmethylated. The DNA methylation track for SQK1-IR in wt Col is produced from BSAS of two overlapping amplicons. Dashed lines align key annotation features of the *SQK1* gene, and the location of the CRISPR gRNA used in the dCas9 experiment is annotated. **b**, RT–qPCR of plants homozygous for the *SQK1* promoter deletion allele *sqk1-1*. The error bar represents the standard deviation of the mean in three biological replicates. **c**, BSAS of plants with and without the SQK1-IR, the homozygous *sqk1-1* deletion and dCas9. The data display is the same as Fig. 1a. The wt Col sample is the same as replicate 2 in Fig. 5c. **d**, Depiction of three EVD TE transgenes. These transgenes share the exact coding sequence of EVD5 (AT5TE20395) lacking any vector-encoded terminator sequence while differing in their upstream elements. These upstream elements include the 35S constitutive promoter (from Fig. 1), no promoter and the T3A terminator. **e**, RT–qPCR of EVD mRNA in T1 transgenic plants indicates that only 35S:EVD has Pol II expression. The error bar represents the standard deviation of the mean of three biological replicates. **f**, BSAS of T1 transformants for each respective EVD transgene in wt Col. The data display is the same as Fig. 1a. The 35S:EVD sample is the same as replicate 1 from Fig. 1a. **g**, Heatmap of small RNA sequencing from single and double EVD transgenic plants. The siRNAs shown were mapped to the entire length of the EVD coding region, not just the region analysed by BSAS in **f.** The non-transgenic wt Col sample is the same as from Fig. 2a, whereas the 35S:EVD wt Col sample (lane 1) is from the T3 generation, to match the same 35S:EVD generation as in the double-transgenic plants (lanes 4 and 5). **h**, BSAS for the 35S:EVD transgene (top) and the second unexpressed EVD transgene (bottom) in the same transgenic individuals. NA, not applicable, as that transgene is not in this plant line. Biological replicates are shown in Supplementary Fig. 8. The data display is the same as Fig. 1a. The wt Col 35S:EVD sample (lane 1) is from the T3 generation, to match the same 35S:EVD generation as in the double-transgenic plants (lanes 4 and 5).

While Pol II cannot substitute for Pol V (Fig. 1), we have identified that Pol II does play a role in making the target locus receptive to the first round of RdDM. While investigating the targeted methylation of the *SQK1* gene (Fig. 5), we observed that the CHH methylation (indicative of RdDM) was not distributed evenly across the region targeted by siRNAs. Instead, methylation peaks upstream of the transcriptional start site near the proximal *SQK1* promoter (Fig. 6a). This pattern of methylation does not correlate with the abundance of siRNAs across this region (Fig. 6a), suggesting that the observed methylation pattern is based on the *SQK1* promoter activity. To test the role of Pol II expression during the first round of RdDM, we used Cas9 to generate a 1,298 bp deletion of the *SQK1* promoter (*sqk1-1*) (shown in Fig. 6a). This severely reduces, but does not completely eliminate *SQK1* expression (Fig. 6b). Where on the *sqk1-1* allele Pol II initiates and the direction of transcription is not known. When the homozygous *sqk1-1* mutation is combined with the SQK1-IR, although the siRNAs still target *sqk1-1*, methylation is substantially reduced (Fig. 6a,c). In a separate test, we generated a second inverted repeat targeting system (as in Fig. 5a) for a different gene that has a specific and limited developmental expression pattern (Supplementary Fig. 8). Our data for both two-component inverted repeat systems (Fig. 6a–c and Supplementary Fig. 8) demonstrate that the strength of RdDM positively correlates with the level of Pol II expression at the target locus.

To decisively test the necessity of Pol II expression in the first round of RdDM, we created two new EVD transgenes that are definitively either expressed or not. These include the ‘EVD-only’ (EVD coding sequence with no promoter) and ‘T3A-EVD’ transgenes (T3A terminator directly 5′ of the EVD coding sequence, to ensure no read-through Pol II transcription) (Fig. 6d). Reverse transcription with quantitative PCR (RT–qPCR) of EVD transgenes confirms that only 35S:EVD has appreciable messenger RNA accumulation (Fig. 6e), although the expression levels generated by the 35S promoter are highly variable due to the nature of T1 transgenesis^31^. We confirmed that RdDM of 35S:EVD is dependent on Pol II expression, as only the expressed transgene version is targeted for methylation (Fig. 6f and biological replicates in Supplementary Fig. 8). The key test was when we combined expressed and unexpressed EVD transgenes in the same genome. We found that whenever the expressed 35S:EVD transgene is present, it produces abundant siRNAs (Fig. 2b and Fig. 6g, lanes 1, 4 and 5), which are incorporated into AGO4 (Supplementary Fig. 6) and drive RdDM to 35S:EVD itself (Fig. 1 and Fig. 6h, lanes 1, 4 and 5). When a second promoterless EVD transgene is introduced into the same plant genome that has 35S:EVD, these unexpressed transgenes do not become methylated (Fig. 6h lanes 4–5, biological replicates in Supplementary Fig. 8). These double-transgenic plants have EVD siRNAs that are incorporated into AGO4 and are competent to perform RdDM (evidenced by the fact that 35S:EVD is targeted by RdDM in the same plant (Fig. 6g, lanes 4–5)). The only difference as to why one transgene is methylated and the other is not, is the Pol II activity at the target transgene. Therefore, Pol II transcription is not sufficient to substitute for the absence of Pol V, but Pol ll activity is necessary to make a locus receptive to the first round of RdDM.

### The role of Pol II in the initiation of RdDM

We aimed to identify the exact function of Pol II during the first round of RdDM. Pol II could be involved in producing a scaffolding transcript for AGO–siRNA interaction. Alternatively, AGO–siRNA complexes are known to interact with single-stranded DNA^32^. Pol II’s function at the target locus could be to open the double-stranded structure of the DNA, allowing for the AGO–siRNA complex to base pair with single-stranded DNA, which fits the theory that RdDM is focused at DNA replication forks^33^. To separate these models, we attempted to increase the RdDM of the low-expressed *sqk1-1* allele by targeting dCas9 to this locus. We used a gRNA to target dCas9 and R-loop formation^34^ to the homozygous *sqk1-1* locus in the presence of the SQK1-IR (see gRNA location in Fig. 6a). This did not result in higher methylation (Fig. 6a,c) as would be expected if DNA opening was the RdDM function of Pol II at the target locus. Instead, methylation decreased (Fig. 6c), as though dCas9 interferes with the low level of Pol II transcription at this locus. Since this dCas9 experiment provided negative results, several alternative interpretations exist. However, this experiment indicates that the function of Pol II at a new RdDM target locus is likely to generate the RNA transcript that acts in AGO–siRNA complementary base pairing.

## Discussion

The roles of RNA polymerase IV and V at silenced TE fragments undergoing the self-reinforcing cycle of RdDM are well described^35^. However, the specific roles of these polymerases and Pol II during the initiation phase of TE and transgene silencing have remained enigmatic. Here we dissect polymerase function using newly integrated transgenes. This strategy permits the discrimination of the first round of RdDM from those already engaged in the RdDM cycle.

We find that Pol V is required for all de novo RdDM, and Pol II cannot substitute for this function. Our data refute the model whereby Pol II transcripts are able to initiate the first round of DNA methylation. The essential function of Pol V during the initiation of RdDM correlates with the recent finding that Pol V is a key factor in the evolutionary repression of TEs in the *Arabidopsis* lineage^36^. Our data also refute the ‘Pol V surveillance’ model, whereby Pol V produces scaffolding transcripts that, when an siRNA is present, will trigger the first round of methylation. It has been recently demonstrated that the artificial tethering of a Pol V-recruitment factor to a genomic locus triggers RdDM^37^. Therefore, if Pol V surveilled everywhere, we would expect to observe spurious low-level methylation across the genome, which is not detected, even in DNA glycosylase mutants that fail to remove DNA methylation from the genome^38^. Although our data refute the Pol V surveillance model, our findings agree with the point that siRNAs are the key determinant to target new RdDM^15^.

**Fig. 7:**
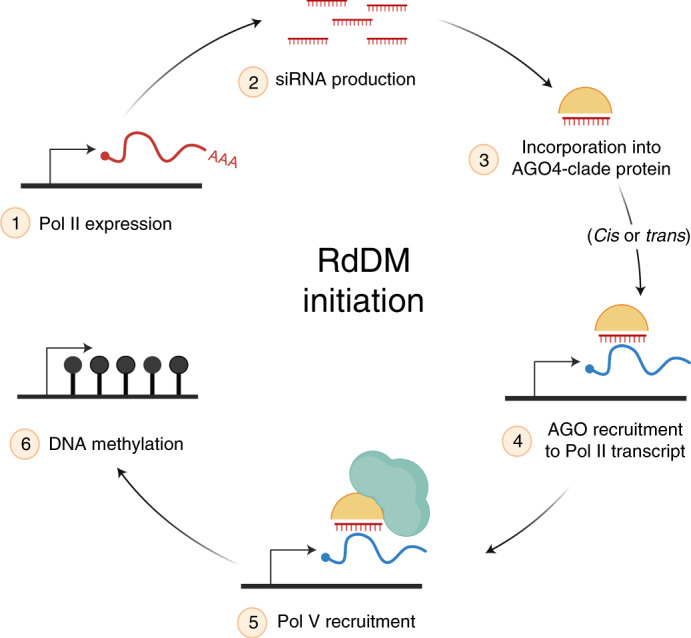
Model of RNA-driven recruitment of Pol V to new RdDM targets. The initiation of RdDM begins with Pol II expression of the unmethylated locus (step 1), followed by siRNA production from that transcript by any member of the DCL family (step 2). The siRNAs are incorporated into a member of the AGO4-clade of proteins (step 3) and this can only target RdDM to complementary regions of the genome that are expressed (step 4). *Cis* action refers to a region that generates siRNAs and targets its own methylation, while in *trans* action the siRNAs are acting on a separate locus. The AGO4 targeting of a Pol II expressed region of the genome results in Pol V recruitment (step 5) and the first round of RdDM (step 6). This final methylated product can then enter the methylation-dependent pathway of continual Pol V recruitment guided by SUVH2/SUVH9. See Discussion for details. Created with BioRender.com.

Our data demonstrate a methylation-independent mechanism for recruitment of Pol V to a new target locus. Previous reports suggested that Pol V is recruited independently of AGO4 and siRNAs, chromatin-associated Pol V recruits AGO4 in a strictly protein-dependent manner^32^, and RdDM would occur only when both Pol V and AGO4 were recruited to a locus^39^. In this previous model, the likelihood that the recruited AGO4 would contain the complementary siRNA that matches the locus of recruitment is low. Our results indicate a more linear ordered pathway of recruitment, with base complementarity of the incorporated siRNA first and independently guiding AGO4 to the chromatin, followed by the recruitment of Pol V. We propose an siRNA-directed pathway of Pol V recruitment in Fig. 7.

Pol II’s function during the first round of RdDM is twofold. First, it is necessary to produce the raw transcripts for siRNA production (step 1, Fig. 7). During the self-reinforcing cycle of RdDM at endogenous silenced TEs, this function is taken over by Pol IV. However, without existing heterochromatic marks to recruit Pol IV, the new region must be transcribed by Pol II to generate siRNAs in *cis* or siRNAs provided from a separate locus in *trans*. We find that in cases where Pol II is generating siRNAs, mutations in the downstream RdDM factors (such as *pol V*, *drm1/2* and *ago4/6/9*) result in the lack of DNA and H3K9 methylation, leading to more Pol II expression and more substrate for the higher accumulation of siRNAs (Fig. 2).

Once the raw transcripts are produced by Pol II, we find that they can be processed by any DCL protein into 21–24-nt siRNAs (step 2, Fig. 7). All these siRNAs sizes, generated by any of the four *Arabidopsis* DCL proteins, are capable and sufficient to target the first round of RdDM. Our data agree with the ‘all hands on deck’ model whereby during the initiation of RdDM all available DCL proteins function to process the large volume of Pol II transcripts, producing 21, 22 and 24-nt siRNAs that are all capable of guiding RdDM^16^. These siRNAs are loaded into an AGO4-clade protein (step 3, Fig. 7), as RdDM function was abolished in the *ago4/6/9* triple mutant. While all DCL and AGO4-clade proteins are sufficient to initiate RdDM, our data indicate that DCL3, 24-nt siRNAs and AGO4 primarily perform this function in wild-type plants.

Second, Pol II action is required downstream of siRNA production. Even when complementary siRNAs and AGO4 are present, the first round of RdDM is dependent on Pol II activity at both transgenes and endogenous genes (Fig. 6). Our data indicate that the role of Pol II at the target locus is to generate the first scaffolding transcript for AGO–siRNA interaction (step 4, Fig. 7). This is supported by the protein–protein interaction of the Pol V-recruitment factor RDM1 with Pol II, AGO4 and DRM2 (ref. ^24^). This function of Pol II suggests that it retains some of its ancestral ability to generate scaffolding transcripts, which has otherwise been subfunctionalized and relegated to Pol V. Even though the Pol II transcript interaction with the AGO–siRNA complex does not result in methylation, it is necessary for the recruitment of Pol V (step 5, Fig. 7) and subsequent RdDM (step 6, Fig. 7). A recent study demonstrated that Pol II’s C-terminal domain can act to recruit more Pol II protein^40^, and during the initiation of RdDM Pol II may act in a similar manner to recruit Pol V, target RdDM and result in the establishment of epigenetic inheritance.

## Methods

### Plant growth and lines

*A. thaliana* plants were grown at 22 °C on Pro-Mix FPX soil in Conviron MTPS-120 growth chambers with 16-h per 200 µmol m^−2^ s^−1^ light. The specific alleles of all mutants are shown in Supplementary Table 1. Inflorescence (flower buds, stages 1–12) tissue was used for all experiments unless otherwise noted. All transgenic material is the first generation (T1) after integration of the T-DNA into the genome unless otherwise stated. All transgenic lines were stably integrated and produced by the Agrobacterium-mediated floral dip method and subsequent selection for Basta- or Hygromycin-resistant plants. Biological replicates are non-overlapping pools of tissue collected from T1 transgenic plants. Double EVD transgenic lines (Fig. 6g,h) were generated by floral dipping T2 Basta-resistant 35S:EVD plants with Hygromycin-resistant EVD-only and T3A-EVD transgenes. Screening and genotyping in the next generation resulted in T3 35S:EVD + T1 EVD-only or T1 T3A-EVD plants. To create the *dcl1/2/3/4* 35S:EVD transgenic plants, we transformed a line that was homozygous *dcl2/3/4* and heterozygous for the *dcl1-9* allele. Resulting transformants were genotyped for DCL1, and divided into those that were homozygous mutants and those that were DCL1 heterozygotes.

### Transgene generation

Transgenes were generated from primers listed in Supplementary Table 1. The EVD coding sequence (At5TE20395) was directly amplified from wt Col DNA using primers containing appropriate plasmid homology for In-Fusion Cloning (Clontech) into their respective digested binary vector backbones. Both pB2GW7 (Basta selection) and pH2GW7 (Hygromycin selection) digested with SpeI/HindIII were used for 35S:EVD, as some mutants that were transformed with this transgene already contain a Basta resistance cassette. pH2GW7 digested with SacI/HindIII was used for EVD only. For T3A-EVD, the T3A terminator and EVD coding sequence were separately amplified and then joined together using overlapping PCR before In-Fusion Cloning into pH2GW7 digested with SacI/HindIII.

The inverted repeat regions of transgenes were synthesized by Thermo Fisher and cloned into the binary vector pEG100 via SpeI/XhoI restriction digest followed by ligation using T4 DNA Ligase (NEB). The SQK1 inverted repeat contains bases AtChr3 3895083:3894528 (order reversed to prevent open reading frame) followed by the PDK intron and then AtChr3 33894528:3895083 (resulting plasmid named pMJS064). The RHD6 inverted repeat was fashioned similarly containing bases AtChr1 24795653:24795105. See Supplementary Table 1 for inverted repeat sequences. A Hygromycin-resistant version of each of these transgenes was created by amplifying the 2× 35S promoter: Hygromycin b phosphotransferase: 35S terminator cassette from the pDIRECT_21B vector using primers from Supplementary Table 1 to add MluI and SacI restriction sites before being digested and ligated into the inverted repeat-pEG100 vectors in place of the Basta cassette.

The *sqk1-1* allele was created using the egg-cell promoter/enhancer CRISPR–Cas9 system described by ref. ^41^. The two-gRNA cassette was created using a four primer overlapping PCR (Supplementary Table 1) to amplify the 20 nt gRNA: U6-26 terminator: U6-29 promoter: 20 nt gRNA from the pCBC-DT1T2 plasmid^42^. This PCR product was cloned via BsaI Golden Gate reaction into a version of the pHEE401E plasmid containing an additional NapA promoter: dsRED: Nos terminator at the MfeI restriction site. The dsRED cassette was amplified out of the Traffic Lines^43^ (primers in Supplementary Table 1) and provides a method for negative selection of the Cas9 transgene. T1 transformants were selected on Hygromycin plates and screened for *SQK1* promoter deletion via PCR (primers in Supplementary Table 1).

To create the dCas9 + SQK1-IR system, the pHEE401E plasmid was digested with NcoI and EcoRI to remove the active Cas9 cassette. The dCas9 sequence was amplified from the pDIRECT_21B plasmid^44^. The Rps5a promoter for dCas9 was synthesized by Thermo Fisher and cloned into the pHEE401E backbone via In-Fusion reaction (Clontech). A gRNA cassette was created and inserted via Golden Gate reaction as described above using primers in Supplementary Table 1. dCas9 targets *SQK1* at the sequence ATACTCAAAAATTAATAGA. The gRNA–dCas9 complex was amplified (primers in Supplementary Table 1) and In-Fusion cloned (Clontech) into a SacI digested pMJS064 (described above) that contains the SQK1-IR. The Rbs terminator for dCas9 was amplified from pHEE401E and In-Fusion cloned into the pMJS064 + dCas9 plasmid following digestion with AvrII (resulting plasmid named pMJS082). The dCas9 construct without the SQK1-IR was created by removing the SQK1-IR from pMJS082 via restriction digest with SpeI and XhoI.

### Expression analysis

RNA was extracted from three biological replicates using TRIzol reagent (Thermo Fisher) and then DNase I treated and cleaned-up with the TURBO DNase DNA-free kit (Thermo Fisher) according to the manufacturer’s protocol. Complementary DNA synthesis was performed using an oligo d(T) primer and Tetro Reverse Transcriptase (Bioline). We performed RT–qPCR using the primers in Supplementary Table 1 and SYBR green supermix. Relative expression was determined using the 2^−∆∆Ct^ method, comparing the gene of interest to a housekeeping control gene (*AXS2* or *ACTIN 2*, Supplementary Table 1). Mean and standard deviations of three biological replicates are calculated by Graphpad Prism and shown as bar graphs with error bars.

### BSAS and analysis

Genomic DNA was treated with RNase A, then cleaned and recovered by phenol chloroform isolation. Then 500 pg–2 µg DNA was treated with sodium bisulfite using the EZ DNA Methylation kit (Zymo Research). Amplicons for sequencing were generated by PCR using degenerate primers (Supplementary Table 1) for each locus using My Taq HS mix (Bioline) and gel purification. For each bisulfite conversion reaction performed, we also performed PCR on a control unmethylated locus (At2G20610) to calculate the conversion rate (see Supplementary Fig. 1 for an example).

To generate amplicon libraries, purified amplicons were pooled in equimolar ratios. Here, 100 ng of purified amplicon DNA in 30 µl volume were used for library preparation with the Illumina Nextera DNA Flex kit. BSAS libraries from December 2019 to present were created using a modified Nextera Flex protocol (first described by ref. ^45^). Per index, 1 µl of Tagmentation (BLT) beads from the Nextera Flex kit was diluted with 19 µl of ultrapure water and combined with 30 µl of sample DNA and 50 µl of laboratory-made 2× tagmentation buffer (20 mM Tris, 20 mM MgCl, 50% DMF) before being tagmented at 55 °C for 15 min. Tagmenation was stopped by adding 20 µl of 0.2% SDS and incubating at 37 °C for 15 min. Beads were washed three times with 100 µl of laboratory-made tagmentation wash buffer (10% PEG8000 and 0.25 M NaCl in TE buffer). Tagmented DNA was amplified directly from the beads via six cycles of PCR amplification using the PrimeSTAR GXL Polymerase kit (Takara) and dual-indexed adapters (Illumina) in 45 µl reactions. Finally, libraries were purified using 81 µl of SPRIselect beads (Beckman Coulter) or Nextera Purification Beads (Illumina), washed twice in 200 µl of 80% EtOH and eluted off beads in 32 µl of ultrapure water.

We sequenced the resulting libraries with 300-nt single-end reads on the Illumina MiSeq platform at the University of Delaware DNA Sequencing and Genotyping Center. Raw reads were trimmed for adapters and mapped to all the amplicons pooled together for sequencing using *methylpy* (https://github.com/yupenghe/methylpy)^46^. Reference DNA sequences have primer sequences removed. The allC files (Data and Code availability) containing methylation data for each cytosine were used as input for Bedtools^47^ to calculate methylation percentage for each locus and plotted using ggplot2 in R. The 95% Wilson confidence intervals were calculated as in ref. ^48^ for error bars. BSAS data of DNA methylation levels were quantified per amplicon as the average methylation at each cytosine sequence context (CG, CHG, CHH). The total number of reads reporting the methylation status of a locus is noted as BSAS coverage in Supplementary Fig. 1. Data were analysed in R and plotted with ggplot2. In the data display, the bar displays the methylation percentage and the error bars represent the 95% confidence interval calculated using the Wilson score interval method. ‘*n*’ denotes the total number of cytosines, which is calculated as the sum of read coverage for all assayed cytosines in an amplicon.

### Bisulfite Sanger sequencing and analysis

Amplicons for Sanger sequencing from Supplementary Fig. 1 were generated as above for BSAS. Purified amplicons were subjected to single colony purification by TOPO TA cloning into pCR4 (Thermo Fisher) and transformation into *Escherichia coli*. Individual colonies were sequenced by Sanger sequencing (Eton Biosciences) and analysed in Kismeth^49^ using default parameters.

### Analysis of whole-genome bisulfite sequencing data

Genome-wide MethylC-seq data are publicly available for wt Col *Arabidopsis* inflorescence^50^. Processed data were downloaded from GEO (GSM2101949). Similar to BSAS analysis, the allC file was used as input for Bedtools^47^ to calculate methylation percentage for each locus shown in Supplementary Fig. 1.

### Determination of RdDM strength

To measure RdDM strength, we calculated the number of consecutive cytosines that are methylated for a given locus per sequencing read. Using methylpy^46^ we isolated the reads that mapped to a specific locus and used these as input for Kismeth^49^. The Kismeth output displays an image of methylation status of each individual cytosine along each individual read, and this was used for image analysis. We used a custom python script (https://github.com/jpeasari/Dot-Plot-Anaysis-OpenCV) to analyse each read in the image, represented by one row of methylation data and determined the longest stretch of consecutive methylated cytosines in each row. We summarized the longest stretch counts for each locus in box plots using ggplot2 in R. Box plots of RdDM strength represent 25th and 75th percentile values with whiskers at the 10th and 90th percentiles, the median is represented by a line and the mean is denoted by a filled circle.

### Small RNA sequencing and analysis

Small RNA was sequenced as in ref. ^17^. Briefly, Trizol reagent (Thermo Fisher) was used to isolate total RNA. The mirVana microRNA isolation kit (Thermo Fisher) was used to enrich small RNAs. The TruSeq Small RNA Library Preparation kit (Illumina) was used to generate libraries and multiplexed for sequencing on a HiSeq 4000, HiSeq X or NextSeq 550 system at the University of Delaware DNA Sequencing and Genotyping Center or Novogene Inc.

Postsequencing, the Illumina universal adapter was removed from the demultiplexed libraries using the fastx toolkit. Total genome matching reads were calculated using bowtie (parameters: -v 0), and this value is used for library depth normalization. The sRNA Workbench^51^ was used to filter out transfer/ribosomal RNA reads, low complexity reads and retain only 18–28-nt small RNA reads that map the *Arabidopsis* Araport11 genome. To map the small RNAs to the genome, ShortStack^52^ was used with parameters:–nohp–mmap f–bowtie_m all–align_only. For assaying siRNAs from a specific transgene, bowtie (parameter: -v 0–best–strata -M 1) was used to map the small RNAs to the full transgene sequence. *Bedtools* was used to count the number of reads mapping a specific locus. In Fig. 3d, replicate 2 of *ago4/6/9* contains the 35S:EVD transgene (from Fig. 2b). The small RNAs mapping the transgene were removed before analysis for Fig. 3d. In Fig. 4a, de novo clusters of 23–24-nt siRNAs were called using ShortStack with a threshold of at least ten raw reads in wt Col and Pol V-dependence was determined by at least a ≥2-fold loss of siRNAs in *pol V* mutants. Conversely, loci that did not lose siRNAs in *pol V* compared to wt Col were categorized as Pol V-independent siRNA loci. ggplot2 in R was used to generate siRNA heatmaps.

### AGO4-incorporated small RNA library preparation and analysis

Frozen inflorescence tissue was ground with liquid nitrogen and resuspended in lysis buffer (50 mM Tris pH 8, 150 mM NaCl, 5 mM MgCl_2_, 10% glycerol, 1% IGEPAL, 0.5 mM DTT, 1 mM PMSF, 1× Roche protease inhibitor cocktail) and homogenized with mixing for 15 min at 4 °C. Lysates were then clarified with a spin. Clarified lysates were combined with 2 μl of AGO4 antibody (Agrisera) or 2 μl of rabbit IgG as a mock-IP (Cell Signaling Technology) and rotated at 4 °C for 1 h. Immune complexes were harvested with 40 μl of Protein G Dynabeads (Thermo), prewashed in 1× TBS, rotating 30 min at 4 °C. Beads and immune complexes were washed three times with 1 ml of cold wash buffer (50 mM Tris pH 8, 150 mM NaCl, 5 mM MgCl_2_, 0.5 mM DTT). Immunoprecipitated small RNA (bound by AGO4) was released from beads and isolated by TRIsure (Bioline) extraction. All RNA recovered from AGO4 IPs went directly into small RNA library preparation.

The small RNA library was prepared using TruSeq Small RNA Library Preparation kit (Illumina) as described above for total small RNAs with the exception of using 14 cycles of PCR amplification. Small RNAs were processed exactly as described earlier and accumulation was calculated in reads per million (RPM) genome-mapped sequenced reads for each size class of small RNAs in AGO4-IP and mock-IP samples. The small RNA enrichment was calculated as the ratio of RPM values for AGO4-IP over mock samples for each size class. This enrichment value is displayed as a heatmap in Fig. 2c and Supplementary Fig. 6.

### Plasmid-safe PCR assay

Frozen inflorescence tissue was ground with liquid nitrogen, and total DNA was purified using the QIAGEN DNeasy Plant Mini Kit. Then 1 μg of the resulting RNased DNA was digested using 1 μl of Plasmid-Safe ATP-dependent DNase (Lucigen) in a 50-μl reaction for 16 h at 37 °C. Digestion was completed by twice adding 1 μl of additional DNase and 1 μl of additional ATP followed by 2 h of incubation at 37 °C for a total of 20 h of digestion. DNase was inactivated at 70 °C for 30 min. Digested and undigested DNA from each line was amplified using PCR primers for the EVD coding region (Supplementary Table 1).

### Western blotting

Frozen inflorescence tissue was ground with liquid nitrogen and resuspended in lysis buffer (50 mM Tris pH 8, 150 mM NaCl, 5 mM MgCl_2_, 10% glycerol, 1% IGEPAL, 0.5 mM DTT, 1 mM PMSF, 1× Roche protease inhibitor cocktail) and homogenized with mixing for 15 min at 4 °C. Lysates were clarified with a spin, combined with 2× loading buffer, denatured and then loaded onto a 4–20% gradient Tris-Glycine gel (Thermo). Protein was transferred from the gel to a polyvinyldifluoride membrane using the BioRad semidry transblot. Membranes were blocked for 1 h at room temperature in 3% milk powder 1× PBS-T. Primary antibodies, which include Pol V (Wierzbicki laboratory), AGO4 (Agrisera) and ACT11 (Agrisera), were all diluted 1:1,000 in 3% milk 1× PBS-T solution and incubated on blots overnight. Washes were performed at room temperature with 1× PBS-T. Antirabbit secondary antibody (Sigma) was used for visualization of Pol V and AGO4, while antimouse secondary (Sigma) was used for ACT11. Blots were visualized using HRP chemiluminescence (Thermo), with exposures ranging from 5 s to 5 min.

### ChIP and qPCR

Nuclei were crosslinked as follows: frozen inflorescences (300 mg per biorep) were ground with liquid nitrogen and resuspend in nuclear isolation buffer (10 mM HEPES, 1 M sucrose, 5 mM KCl, 5 mM MgCl_2_, 0.6% Triton X-100, 0.4 mM PMSF, 1× Roche protease inhibitor cocktail) and homogenized with mixing for 15 min at 4 °C. Methanol-free formaldehyde (Pierce) was added to a final concentration of 1% with end-over-end mixing for 15 min at room temperature. Formaldehyde crosslinking was quenched with glycine (125 mM), rotating 5 min at room temperature. Crosslinked nuclei were then filtered through two layers of Miracloth to remove large particles before centrifugation at 3,000*g* for 15 min at 4 °C. The resulting nuclear pellet was resuspended in wash buffer (10 mM Tris pH 8, 0.25 M sucrose, 10 mM MgCl_2_, 1 mM EDTA, 1% Triton X-100, 1× Roche protease inhibitor cocktail) and nuclei were cleaned and pelleted at 12,000*g* for 10 min at 4 °C. The final clean nuclear pellet was resuspended in 1 ml of nuclear lysis buffer (20 mM Tris pH 8, 2 mM EDTA, 0.1% SDS, 1 mM PMSF, 1× Roche protease inhibitor cocktail) and sonicated on the Covaris E220 (150 W peak power, 20% duty factor, 200 cycles per burst, 6 min). Insoluble debris was removed from the sonicated soluble chromatin by centrifugation at 12,000*g* for 10 min at 4 °C. Then 30 µl of 5 M NaCl and 20 µl of 30% Triton X-100 were both added to 920 µl of sonicated chromatin to sequester SDS before IP. Then 3% input by volume was set aside for each sample, and the remaining volume of sonicated chromatin was divided evenly among IPs and IgG negative controls for overnight IP with respective antibodies or IgG.

Pol V (Lagrange laboratory antibody) and Ser5P Pol II (Abcam) ChIPs were performed with 2 µl of antibody, whereas AGO4 (Agrisera) and H3K9me2 (Abcam) ChIPs used 5 µl of antibody per IP. Each experiment used the same volume of rabbit IgG as a negative control (Cell Signaling Technology). Immune complexes were collected using 40 µl of washed protein A/G magnetic beads (Pierce), rotating at 4 °C for 2 h. After collecting immune complexes, beads were washed at 4 °C using one rinse and two 5-min washes of each of the following buffers: low salt (150 mM NaCl, 0.1% SDS, 1% Triton X-100, 2 mM EDTA, 20 mM Tris pH 8), high salt (500 mM NaCl, 0.1% SDS, 1% Triton X-100, 2 mM EDTA, 20 mM Tris pH 8), LiCl buffer (250 mM LiCl, 1% Igepal, 1% sodium deoxycholate, 1 mM EDTA, 10 mM Tris pH 8) and TE + 0.1% Igepal. Chromatin was eluted from the beads using 250 µl of elution buffer (1% SDS, 0.1 M NaHCO_3_) at 65 °C for 15 min with agitation. Overnight reverse crosslinking was accomplished with the addition of 20 µl of 5 M NaCl at 65 °C for all samples including inputs. Proteinase K digestion was performed by the addition of 10 µl of 0.5 M EDTA, 20 µl of 1 M Tris pH 7, 20 μg Proteinase K (Thermo Scientific) and 50 ng RNase A, incubating at 42 °C for 1 h. DNA was then purified using DNA Clean and Concentrator-25 columns (Zymo Research) and eluted in 100 µl of elution buffer.

qPCR was performed using primers in Supplementary Table 1 and Sso Universal SYBR (BioRad). Percentage input was calculated by first normalizing the Ct values of the diluted input samples to 100% input by the following calculation: Ct_100%_ = Ct_diluted_ − (log(dilution factor, 2)). Then the IgG and IP Ct values were normalized to this new 100% input Ct value using the 2^−∆∆Ct^ method, which represents percentage input. For Pol V and AGO4 ChIP, the *pol V* mutant and *ago4/6/9* mutants (*ago4-4* allele, a complete null) were, respectively, used to calculate background levels for each of these antibodies. These background levels were averaged for each PCR target. They were then subtracted from the percentage input values of each of the other genotypes. Mean and standard deviations of the biological replicates are shown as bar graphs with error bars.

### ChIP–seq and RIP-seq data analysis

Raw reads were downloaded from National Center for Biotechnology Information (NCBI) GEO (GSE52041 and GSE70290), trimmed for adapters and mapped to the Araport11 *Arabidopsis* genome. The ChIP–seq reads were mapped using *Shortstack* (parameters:–nohp–mmap f–bowtie_m all). The RIP-seq reads were mapped using Soapsplice v.1.10 (ref. ^53^) using parameters: -t 10300 (maximum distance between two segments) as mentioned in the original study^9^. For Fig. 3a, reads were counted using *Bedtools* for each individual locus. To determine enrichment, the normalized ratio of IP over mock sample reads was calculated (for both ChIP–seq and RIP-seq). In eukaryotes, organelle-to-nucleus DNA transfer is known to occur, which has resulted in some mitochondrial genes being duplicated and part of the nuclear genome. To avoid such genes causing an artefact of Pol V enrichment in the negative control dataset, we analysed only those mitochondrial genes that do not have any similarity to nuclear genes.

In Fig. 3c, genome-wide Pol V enriched loci were determined using the *Macs2* ChIP peak caller^54^. Pol V fold enrichment was calculated using the ratio of IP over mock samples. The two replicates were averaged for wt Col and *suvh2/suvh9* mutant. The loci that had ≥2-fold Pol V enrichment in wt Col and only background levels of Pol V enrichment in *suvh2/suvh9* were categorized as SUVH2/SUVH9 fully dependent Pol V loci. The loci that retained at least ≥2-fold Pol V enrichment in *suvh2/suvh9* mutants were categorized as SUVH2/SUVH9 partially dependent Pol V loci. In Fig. 4c, AGO4 enrichment was calculated as a ratio of IP over mock normalized read accumulation. For genome-wide analyses, the two biological replicates were averaged, whereas the replicates are also displayed individually in Fig. 3a.

### Reporting Summary

Further information on research design is available in the Nature Research Reporting Summary linked to this article.

## Supplementary information

Supplementary Information Supplementary Figs. 1–8 and Table 1.

Reporting Summary

Supplementary Data 1 BSAS processed data of DNA methylation levels.

Supplementary Data 2 Sanger sequencing of bisulfite converted DNA.

## Data Availability

Raw Illumina sequencing data produced for this study is available without restriction from NCBI as GSE165575. Additional small RNA datasets were downloaded from GSE118705. Genome-wide MethylC-seq data is publicly available for wt Col *Arabidopsis* inflorescence^50^. Processed data were downloaded from GEO (GSM2101949). ChIP–seq and RIP-seq reads were downloaded from NCBI GEO (GSE52041 and GSE70290). Processed BSAS data of DNA methylation levels are available as Supplementary Dataset 1. Sanger sequencing results are available as Supplementary Dataset 2. Biological materials can be obtained from the corresponding author without restriction.

## Code availability

The custom script used to generate RdDM strength plots is available on Github at https://github.com/jpeasari/Dot-Plot-Anaysis-OpenCV.
