# Supplementary material for: An siRNA-guided ARGONAUTE protein directs RNA polymerase V to initiate DNA methylation
Source: Nat Plants. 2021 Nov 8;7(11):1461–74. doi: 10.1038/s41477-021-01008-7 (PMC8592841; doi:10.1038/s41477-021-01008-7)
Supplement: Supplementary file 1 — Supplementary Figs. 1–8 and Table 1. [file 41477_2021_1008_MOESM1_ESM.pdf]

nature portfolio

https://doi.org/10.1038/s41477-021-01008-7

Supplementary information

An siRNA-guided ARGONAUTE protein directs RNA polymerase V to initiate DNA methylation

In the format provided by the authors and unedited

Suppplemental Figure 1

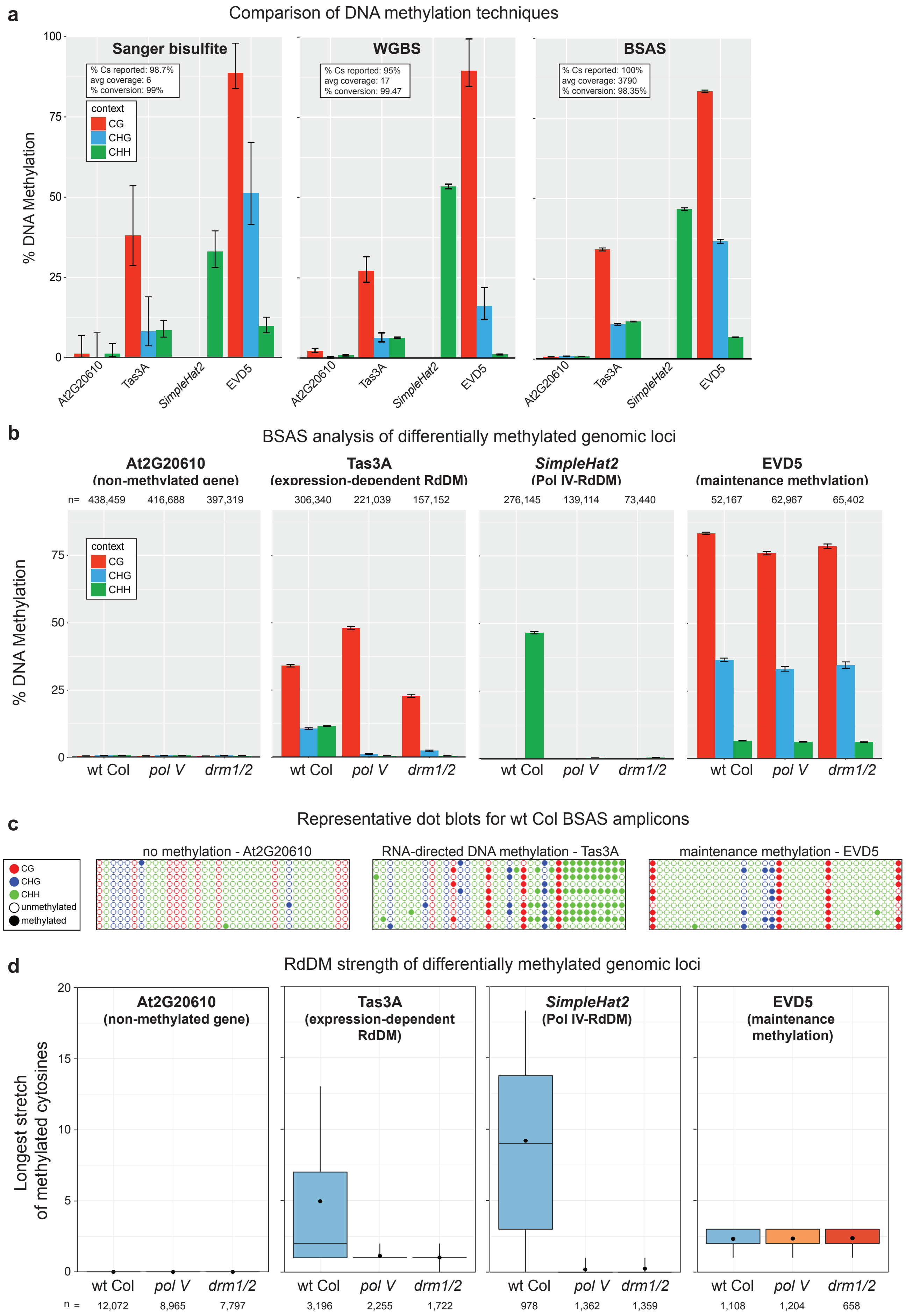

Supplemental Figure 1 - BSAS and RdDM strength validation

(**a**) Comparison of three DNA methylation assays (Sanger sequencing of bisulfite converted DNA, whole genome bisulfite sequencing (WGBS), and bisulfite amplicon sequencing (BSAS)) at endogenous loci representing four different methylation mechanisms and scenarios: a non-methylated gene (At1G20610), an expression-dependent RdDM locus (Tas3A), a Pol IV-RdDM TE (*SimpleHat2*), and a maintenance methylation TE (the endogenous EVD5 - At5TE20395). The amplicons interrogated by Sanger sequencing are the same as in BSAS. The bar displays the methylation percentage and the error bars represent the 95% confidence interval calculated using the Wilson Score Interval method. BSAS results in much tighter confidence intervals than either Sanger sequencing or WGBS. The bisulfite conversion rate is empirically determined by assaying the methylation of the un-methylated genic locus At2G20610 for each technique. The *SimpleHat2* amplicon only has CHH methylation data, because it lacks CG and CHG cytosine contexts.

(**b**) BSAS of four loci in wt Col, *pol V* and *drm1/2* plants confirms that BSAS is appropriate for assaying DNA methylation at all types of methylated loci from part A. Tas3A and *SimpleHat2* are targeted by RdDM and therefore are dependent on Pol V and DRM1/2 for DNA methylation, while the endogenous EVD5 element undergoes maintenance methylation and is therefore not dependent on these RdDM factors. The data display is the same as part A. n = the number of total cytosines assayed for each amplicon.

(**c**) A snapshot of the dot blot analysis of DNA methylation data produced for 10 individual sequences using *Kismeth* (1). Circles represent the cytosines in the DNA sequences, and filled circles are methylated cytosines. The circle color refers to the cytosine sequence context. The signature of RdDM is horizontal stretches of consecutively methylated cytosines as seen for Tas3A, but not for the maintenance methylation locus of endogenous EVD5.

(**d**) Box plots of RdDM strength summarize the number and length of methylation stretches on individual sequenced reads. The endogenous EVD5 element that undergoes maintenance methylation (see part B-C) is an example of a region that has DNA methylation present but has low RdDM strength. All box plots in this work represent 25th and 75th percentile values with whiskers at the 10th and 90th percentile, the median is represented by a line, and the mean is denoted by a filled circle. N = the number of sequenced reads that contribute to the box plot data for each sample.

Supplemental Figure 2

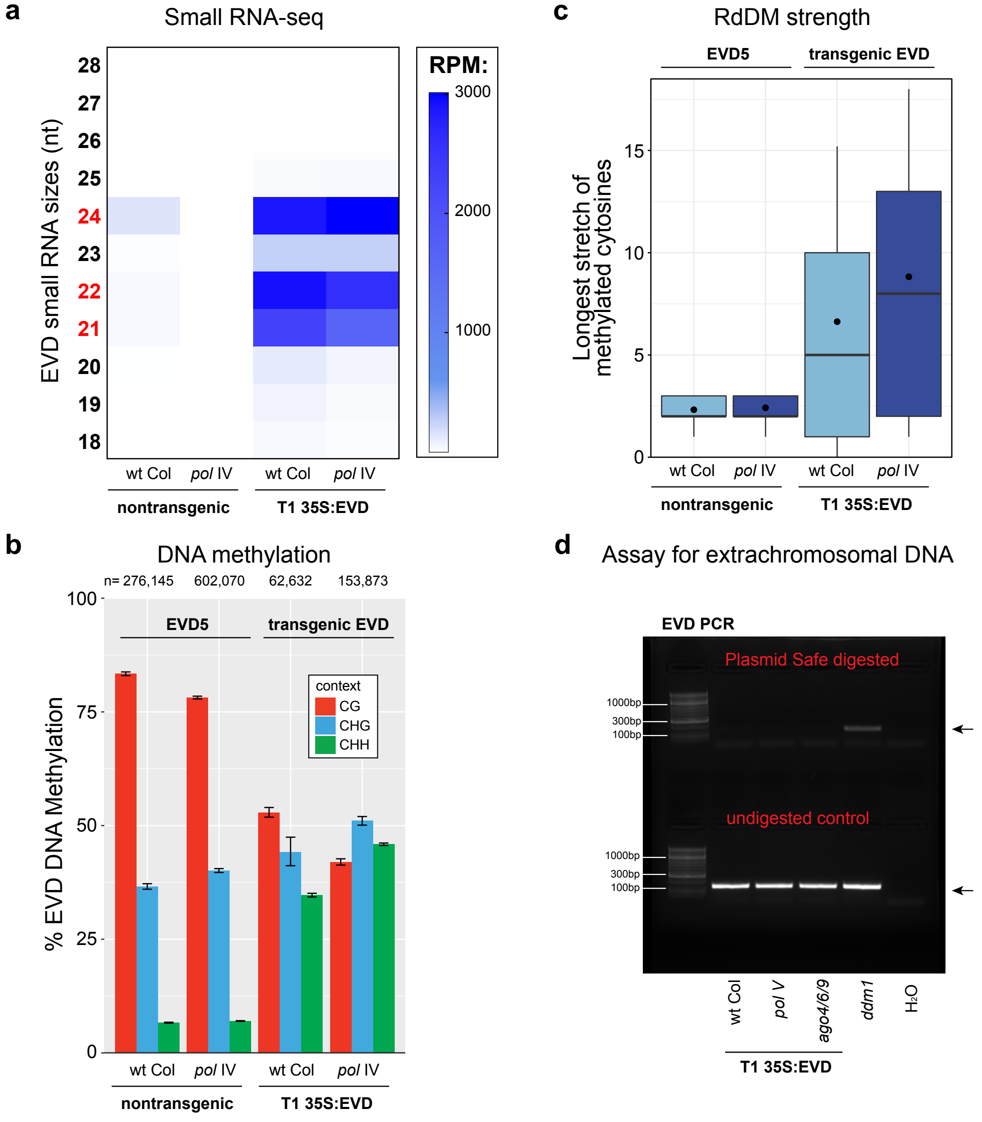

Supplemental Figure 2 - The 35S:EVD transgene is not influenced by siRNAs generated by endogenous EVD elements and is not mobile

(**a**) EVD small RNA accumulation from non-transgenic wt Col and *pol IV* mutants, as well as both these lines with the 35S:EVD transgene. There is a low level of Pol IV-dependent EVD siRNAs in non-transgenic wt Col, while the siRNAs from 35S:EVD are not Pol IV-dependent. The 35S:EVD and non-transgenic samples in wt Col are the same as from Figure 2A, and the *pol IV* 35S:EVD sample is the same as in Figure 2B.

(**b**) BSAS of the endogenous EVD5 element and 35S:EVD. Pol IV is not responsible for the methylation of either locus. The data display is the same as Supplemental Figure 1A. The wt Col 35S:EVD sample is replicate 1 from Figure 1A, whereas the *pol IV* 35S:EVD sample is the same sample as in Figure 2A.

(**c**) Box plots of RdDM strength for the lines from B. This analysis demonstrates that 35S:EVD is undergoing expression-dependent initiation of DNA methylation only. It is not being targeted in *trans* from endogenous EVD via an identity-based mechanism of DNA methylation through Pol IV-dependent siRNAs. Therefore, 35S:EVD represents a transgene that is efficiently and reproducibly targeted in *cis* for *de novo* expression-dependent DNA methylation. Box plot percentiles are the same as in Supplemental Figure 1C.

(**d**) PCR amplification of Plasmid Safe DNase-digested DNA from 35S:EVD T1 lines (wt Col, *pol V*, and *ago4/6/9*) and a nontransgenic *ddm1* (F6) positive control. Undigested control DNA is shown in the bottom row. Plasmid Safe DNase digests genomic (linear) DNA leaving only circular, extrachromosomal DNA available for amplification (2). Only the *ddm1* positive control, in which endogenous EVD has been previously shown to transpose (3), has evidence of mobility. These results demonstrate that the 35S:EVD transgenic lines contain no extrachromosomal copies of either 35S:EVD or endogenous EVD. Arrows represent expected size of EVD amplicon. EVD primers amplify both endogenous EVD and 35S:EVD. This analysis was performed once.

Supplemental Figure 3

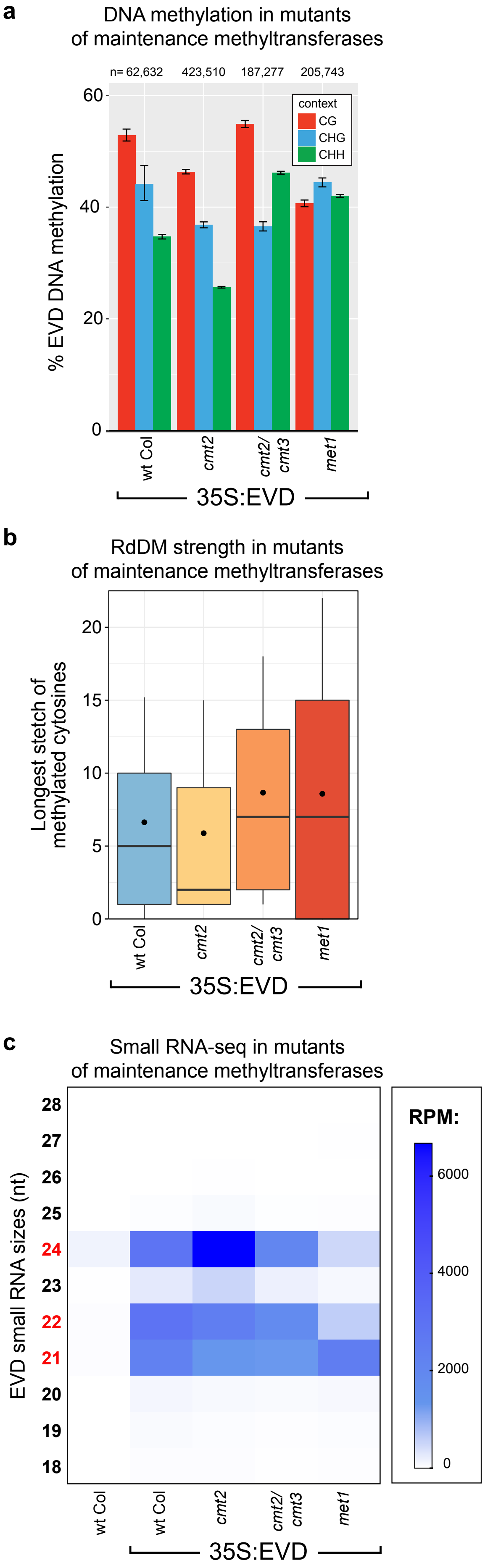

Supplemental Figure 3 - Methylation of 35S:EVD is not dependent upon maintenance methylation factors

(**a**) BSAS of T1 35S:EVD in mutants of maintenance DNA methylation factors. The 35S:EVD CG, CHG and CHH methylation levels in these mutant combinations are targeted by RdDM and established by the DRM1/ DRM2 DNA methyltransferase proteins (Figure 2A). The data display is the same as Supplemental Figure 1A. The wt Col 35S:EVD sample is replicate 1 from Figure 1A.

(**b**) Box plots of RdDM strength in the same lines as part A. Box plot percentiles are the same as in Supplemental Figure 1C.

(**c**) EVD siRNA accumulation in the same lines as part A-B.

Supplemental Figure 4

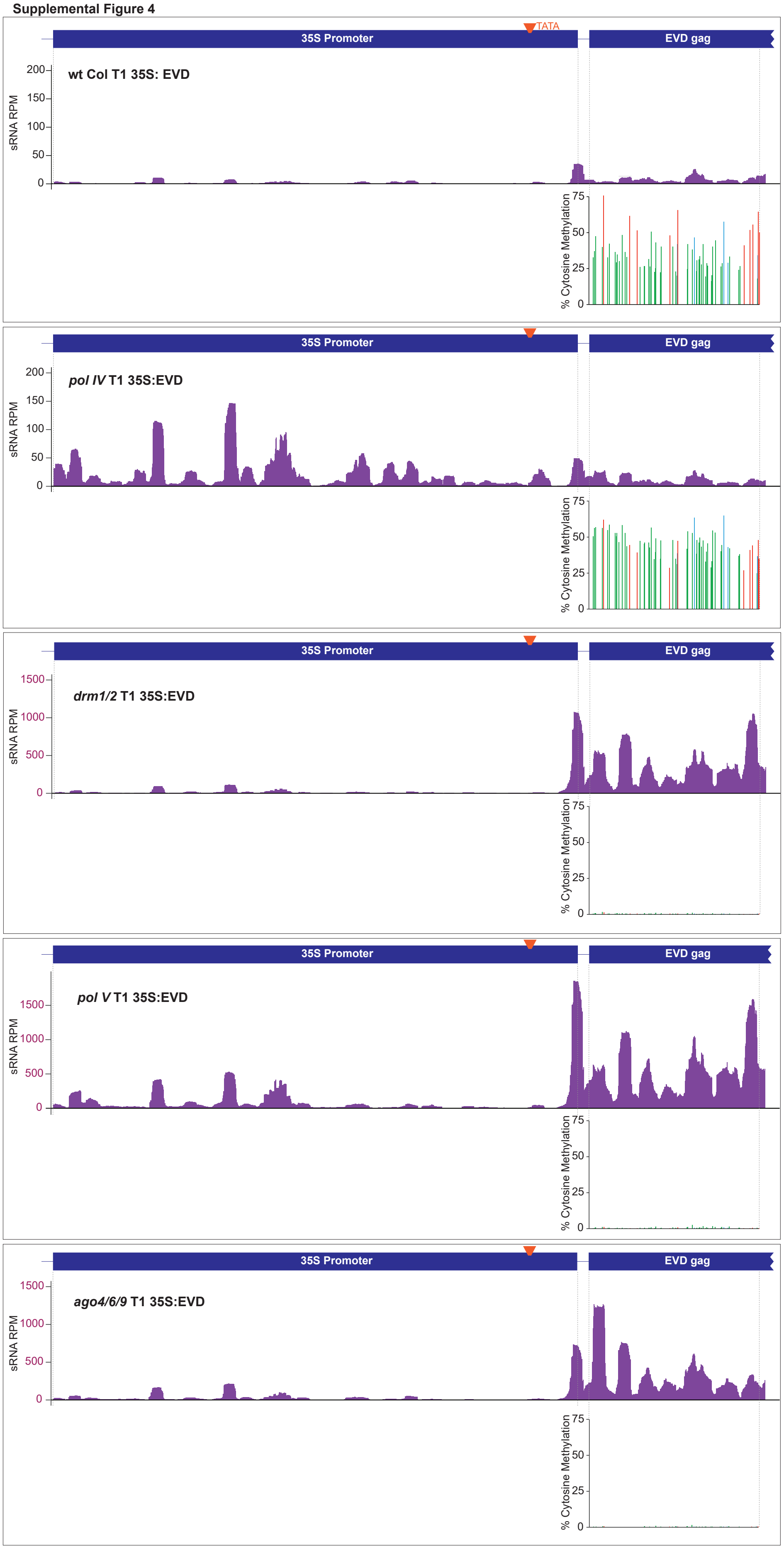

Supplemental Figure 4 - Track images of DNA methylation and siRNA accumulation

DNA methylation and siRNAs (combined 21, 22 and 24 nt) aligned to the 35S:EVD transgene. 35S promoter, TATA box, and EVD coding region are annotated. The siRNA alignments for T1 35S:EVD in *pol V, ago4/6/9*, and *drm1/2* have a larger Y-axis to accommodate for the increased siRNA production in these lines. BSAS methylation data from EVD amplicons is shown below each corresponding siRNA track and aligned to the primer set used for amplification. As shown, despite high levels of siRNA production in the T1 35S:EVD lines *pol V, ago4/6/9,* and *drm1/2*, there is no methylation for the assayed EVD region of the 35S:EVD transgene.

Supplemental Figure 5

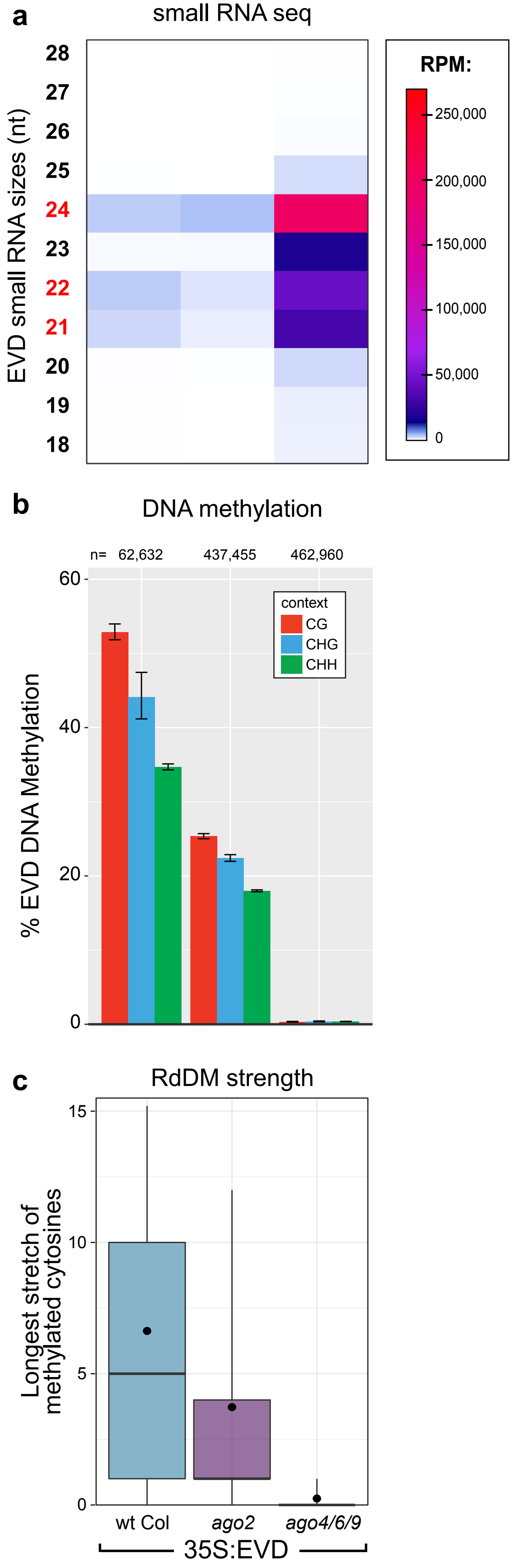

Supplemental Figure 5 - Analysis of *ago2* mutant plants during the first round of RdDM
(**a**) EVD siRNA accumulation of the T1 35S:EVD transgene in *ago2* mutants and mutants of the *ago4/6/9* line, which is shown for comparison.
(**b**) BSAS of T1 35S:EVD in *ago2* mutants. The data display is the same as Supplemental Figure 1A. The wt Col 35S:EVD sample is replicate 1 from Figure 1A.
(**c**) Box plots of RdDM strength in the same lines as part B. Box plot percentiles are the same as in Supplemental Figure 1C.

Supplemental Figure 6

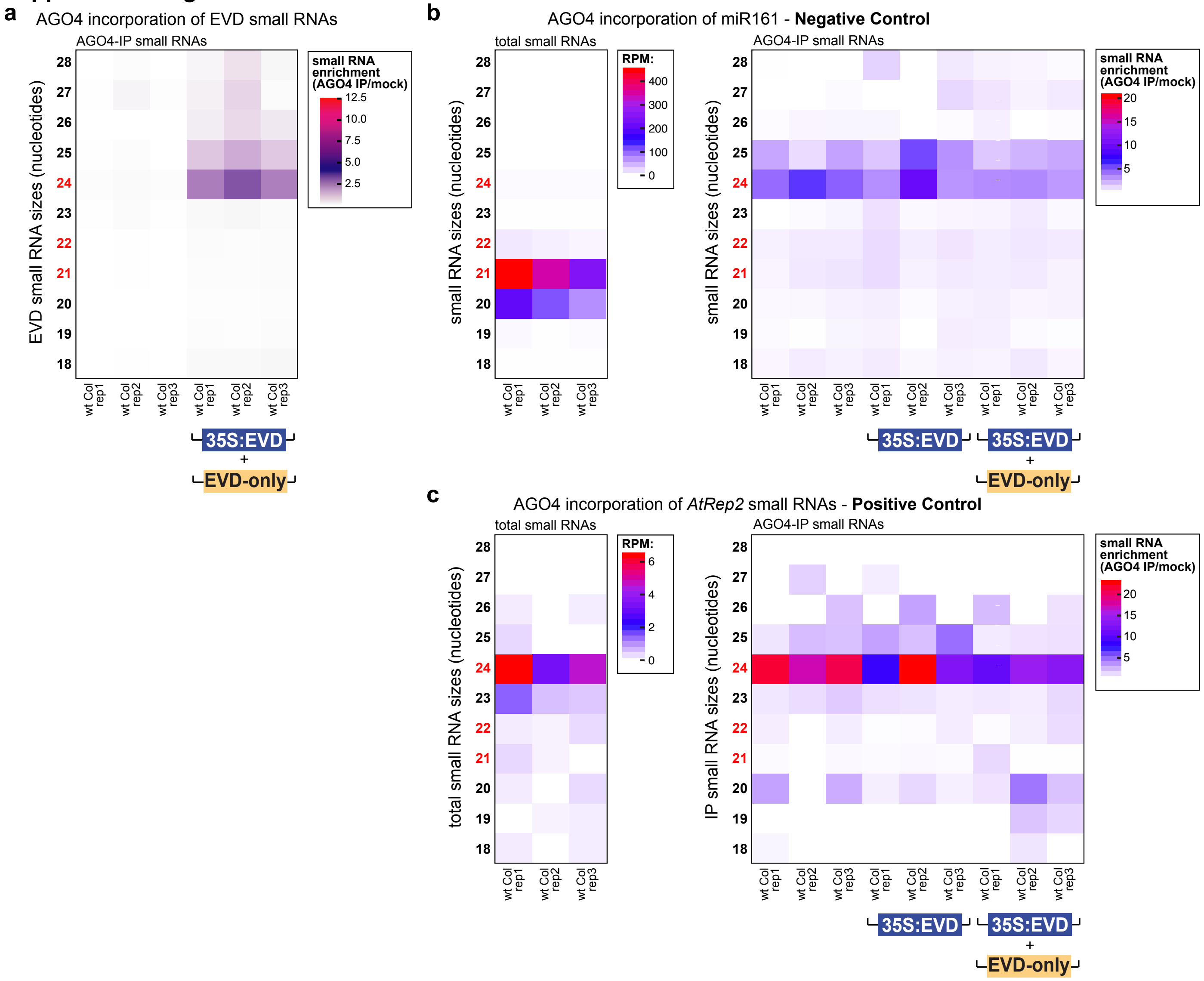

Supplemental Figure 6 - AGO4-IP small RNA sequencing

(**a**) Heatmap of EVD small RNA enrichment in AGO4 for plants from Figure 6G, for plants containing both the 35S:EVD transgene and the no-promoter EVD-only transgene. Enrichment is calculated as the ratio of small RNA accumulation (RPM) in AGO4-IP over mock-IP samples for each size class of small RNA.

(**b**) Negative control demonstrating the quality of AGO4 enrichment analysis for samples from Figure 2C and Supplemental Figure 6A. Heatmap of total small RNAs (RPM) is shown on the left whereas AGO4-IP small RNA enrichment is shown on the right side. Only small RNAs mapping to the miR161 region are displayed. miR161 accumulates in the cell as a 20-21 nt microRNAs (left), and this size class is not incorporated into AGO4 (right). Instead, low levels of a 24-25 nt version of miR161 are incorporated into AGO4.

(**c**) Positive control demonstrating the quality of AGO4 enrichment analysis for samples from Figure 2C and Supplemental Figure 6A. The positive control *AtRep2* is a TE that generates 24 nt siRNAs (see total small RNAs heatmap on the left), which are incorporated into AGO4 (see AGO4-IP enrichment heatmap on the right).

Supplemental Figure 7

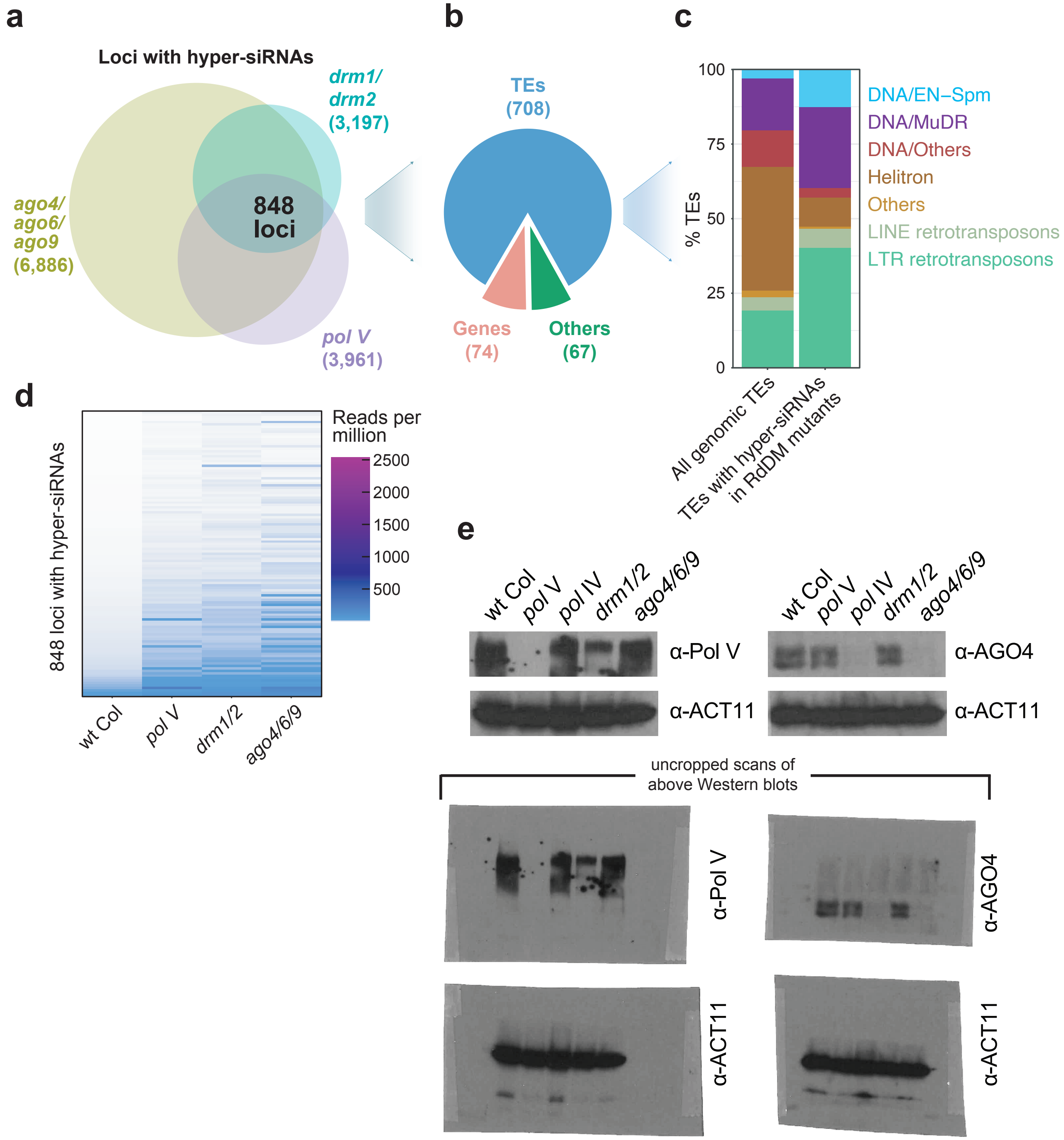

Supplemental Figure 7 - siRNA and protein accumulation in mutants of downstream RdDM factors

(**a**) Venn diagram of loci with at least a 2-fold increase in siRNA production (combined 21, 22 and 24 nt) in *pol V, drm1/2* and *ago4/6/9* mutants. 848 loci have hyper-accumulation of siRNAs in all three mutant combinations.

(**b**) Pie chart of the annotations of the 848 regions.

(**c**) Distribution of TE superfamily annotations of the 708 TE regions form part B.

(**d**) Heatmap of the siRNA accumulation (combined 21, 22 and 24 nt) in 848 loci with increased siR-NAs from part A.

(**e**) Western blots of Pol V and AGO4 protein accumulation in the mutant genotypes used in ChIP experiments. ACT11 protein accumulation is shown as a loading control. All antibodies were used at 1:1000 dilution for blotting. This analysis was performed once.

Supplemental Figure 8

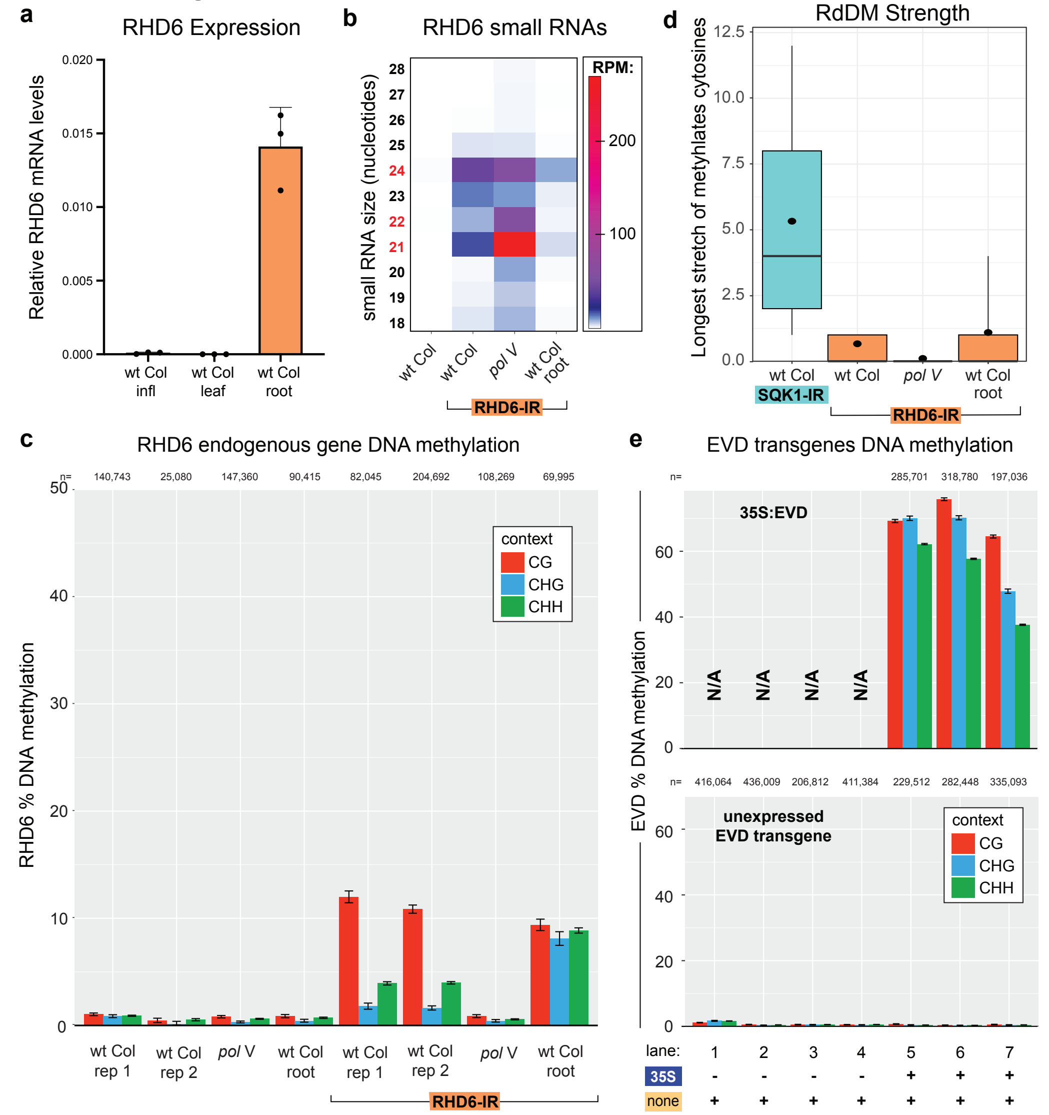

Supplemental Figure 8 - Targeting RdDM to a dynamically expressed gene and unexpressed transgene

(**a**) mRNA accumulation of the ROOT HAIR DEFECTIVE 6 (RHD6, At1G66470) gene measured by qRT-PCR. The RHD6 gene is expressed only in the root hairs and developing embryo (4,5), and not in the inflorescence (Infl) tissue used in our other experiments. The error bar represents the standard deviation of the mean between 3 biological replicates per tissue.

(**b**) Heatmap of small RNA sequencing in T1 transformants. The endogenous RHD6 gene does not produce siRNAs, and upon the addition of the 'RHD6-IR' transgene in the T1 generation, 21-24 nt siRNAs accumulate from the IR transgene.

(**c**) BSAS for T1 plants with the RHD6-IR. The IR siRNAs target a low level of methylation at the RHD6 endogenous gene. This methylation is dependent on Pol V, demonstrating that it underwent RdDM. The higher level of CG methylation compared to CHG and CHH in the inflorescence tissue suggests that this methylation is being maintained, rather than actively targeted as in the root sample that shows equal levels of methylation at all cytosine sequence contexts. The data display is the same as Supplemental Figure 1A.

(**d**) Box plots of RdDM strength for transgenic plants from C. The strength of RdDM is low in the inflorescence tissue analyzed compared to the SQK1-IR system. In inflorescence tissue, the low RdDM strength demonstrates that the unexpressed RHD6 is not actively undergoing RdDM in the inflorescence tissue. Rather this methylation is being maintained from RdDM earlier in development, likely in the embryo. Although the average is similar between inflorescence and root, the 90th percentile whisker shows that there are some sequenced reads from root undergoing RdDM at similar levels as the SQK1-IR, even with fewer IR-derived siRNAs accumulating in the root (part B). Box plot percentiles are the same as in Supplemental Figure 1C.

(**e**) Biological replicates of unexpressed EVD transgenes from Figure 6H. BSAS for the 35S:EVD transgene (top) and the second unexpressed EVD transgene (bottom) are from the same transgenic individuals. N/A = not applicable, as that transgene is not in this plant line. The data display is the same as Supplemental Figure 1A. All data represented here are additional biological replicates of data shown in Figure 6H lanes 2 and 3. No samples are duplicated replicates from Figure 6H.

Supplemental Table 1. Primer sequences and alleles used in this report

Primers and sequences used in this report

Figure	Experiment	Target	Forward primer sequence	Reverse primer sequence
1A	Cloning 35S:EVD	EVD5 - At5TE20395	AGGCGGCCGCACTAGTATGGAGACTTCTCAAAAGATGATTAC	AGCTCAAGCTAAGCTTTCAAGAGTGAGATAGATCCACAAG
1A/B, 2A/F, 6F/H, S2B/C, S3A/B, S4, S5B/C, S8E	BSAS	EVD coding region (35S:EVD)	GGAAGTTYATTTYATTTGGAGAGG	TAATCRCCTTCTTCACTTCAAACAC
1A/B, 2A/F, 5C/D, 6C/F/H, S1A/B/C/D, S2B, S3A, S4, S5B/C, S8C/D/E	BSAS	control gene At2g20610	GTTGYTGATTATATGAAYYGAGATYTT	TTAATTACAACCATARCCACARTRTTCTC
2D/E, 3B, 4D/E	ChIP: H3K9me2, Pol II, Pol V, AGO4	EVD coding region	CTATTCTAGTCGACCTGCAGGCG	AAGGACAGCTTGATCCTCTTGG
2D	ChIP: Pol II	SimpleHat2	AACTCAACCCATGAACCCTAATG	CCATTAACCATTACAACCCAACTC
2D/E, 3B, 4D/E	ChIP: H3K9me2, Pol II, Pol V, AGO4	ASX2	AGGCAACCCAAACAATGAAGTG	GATCGTTGGACTCTCGATAGCTG
2E	ChIP: H3K9me2	*Athila6* TE	AGAAAGAAGAAGGCTGCACTCG	CACCGCTCTGATGATACTCGAC
3B, 4D/E	ChIP: Pol V, AGO4	positive control At5g52070	CATCTGATTCTTAACACCACCTACTCA	ATGTCCTGAGCTGCCACGTT
5A	Cloning SQK1-IR	Full sequence synthesized by Thermo Fisher	35S Pro...CGCTCGAGCAGAGAGATCAA...SQK1...ACGAGCCCTTGGTAAGGAAATA…PDK<br>Intron...TTGGTCTAGAGATTTCGTCTAGATCGTTCA… SQK1...ACTAGTCCCTAGAGTCCTGCTT…OCS TERM	
5C/D, 6A/C	BSAS	SQK1 amp 1	TGTTGGGAAYTTAAAGGATTA	CTTTCTCARAARCARATTTCACCAC
5A, S8	Creating Hygro-IRs	35S-HygroR-35ST	AATTTTCGCGTGAAACCTGTCGTGCCAGC	TAAAATGAGCTCTAACACATTGCGGACGTTTTT
6A	BSAS	SQK1 amp 2	AAAAYYAAAAGTAAAGTTTAATTAG	TTCCATTCCCRTCTATTAATTTTT
6A/C	BSAS	*sqk1-1* deletion allele	AGAAGATGAAGATTYATTGGGTTTG	CTTTCTCARAARCARATTTCACCAC
6B	qRT-PCR	SQK1	TCACTGTGCTTAAGGGCGTT	TGCAATGTCAGGGGCGTAAG
6B/E	qRT-PCR	control gene ASX2	AGGCAACCCAAACAATGAAGTG	GATCGTTGGACTCTCGATAGCTG
6A/B/C	Creating gRNAs for *sqk1-1* (4 primers)	SQK1 gRNAs amplified from pCBC-DT1T2	ATATATGGTCTCGATTGATATGCGTTATCTGCCACGGTT<br>TGATATGCGTTATCTGCCACGGTTTTAGAGCTAGAAATAGC	AACTGCATATGTGGTAGAGACCCAATCTCTTAGTCGACTCTAC<br>ATTATTGGTCTCGAAACTGCATATGTGGTAGAGACCCAA
6A/B/C	Cloning NapP:dsRED:NosT	Nap Promoter/Nos Terminator	CTTCATCGGTGATTGATTCCTTTAAAGACTTAT	GATCTAGTAACATAGATGACAC
6A/B/C	Adding MfeI ends to NapP:dsRED:NosT	Nap Promoter/Nos Terminator	AAAAAACAATTGCTTCATCGGTGATTGA	AAAAAACAATTGGATCTAGTAACATAGATG
6A/B/C	Genotyping for *sqk1-1* deletion	SQK1 promoter	GAGAAGCCAGTCCCAACAGG	GGGTCTCGGTCGTGAAGAAG
6A/B/C	dCas9 Cloning from pDIRECT	dCas9	ATGGATAAGAAGTACTCTATCGGACTCG	TCAAACCTTCCTCTTCTTCTTAGGATCAG
6A/B/C	Creating gRNAs for dCas9 targeting (4 primers)	SQK1 (F) and RHD6 (R) gRNAs amplified from pCBC-DT1T2	ATATATGGTCTCGATTGATACTCAAAAATTAATAGAGTT<br>TGATACTCAAAAATTAATAGAGTTTTAGAGCTAGAAATAGC	AACCCACCGGCATAAGCTGCATCAATCTCTTAGTCGACTCTAC<br>ATTATTGGTCTCGAAACCCACCGGCATAAGCTGCATCAA
6A/B/C	In Fusion Primers for gRNAs + dCas9	dCas9 into pMJS064	CGCCGAATTAATTCGAGCTCCTAGGTTAATCACCACCGAGCTC	AAATTATCAGATCCGAGCTCGTAAAACGACGGCCAGTGC
6A/B/C	In Fusion Primers for Rbsc Term	Rbsc Term into pMJS064 + dCas9	TGGTGATTAACCTAGGAGCTTTCGTTCGTATCATCGG	GAATTAATTCGAGCTCCTAGCAATTGGCAAGTCATAAAATGCA
6D	Cloning EVD-only	EVD5 - At5TE20395	ATGATAATTCGAGCTCATGGAGACTTCTCAAAAGATGATTAC	AGCTCAAGCTAAGCTTTCAAGAGTGAGATAGATCCACAAG
6D	Cloning T3A-EVD	T3A terminator	ATGATAATTCGAGCTCCAGGCCTCCCAGCTTTCG	CTTTTGAGAAGTCTCCATCTCGACACAAAAAGCCTATACTGTAC
6D	Cloning T3A-EVD	EVD5 - At5TE20395	ATGGAGACTTCTCAAAAGATGATTAC	AGCTCAAGCTAAGCTTTCAAGAGTGAGATAGATCCACAAG
6E, S2D	qRT-PCR and Plastmid Safe PCR	EVD coding region	ACAAAAACGGTGTTGTGTGGTC	AAGGACAGCTTGATCCTCTTGG
6F/H, S8E	BSAS	EVD coding region (EVD-only & T3A-EVD)	TAATGGTTTYTGAYGTATGTGYTTAGYT	TAATCRCCTTCTTCACTTCAAACAC
S1A/B/C/D	Sanger bisulfite sequencing & BSAS	Tas3A	GATTATTATAGTTGTAAAGAGTAA	CAACCATACATCAATAACAAACAA
S1A/B/D	Sanger bisulfite sequencing & BSAS	SimpleHat2	AAAAAGTGGAATYYGTAAATATYTTAAGTTTGATG	TATTTTCATATTRTCAAAAAAACRTCTTCTTTCTC
S1A/B/C/D, S2B/C	Sanger bisulfite sequencing & BSAS	EVD5	AGAGYATAAGATYATGTYTTTATG	TAATCRCCTTCTTCACTTCAAACAC
S8	Cloning IR-RHD6	Full sequence synthesized by Thermo Fisher	35S Pro...CGCTCGAGATGTCAGAAGAAGCCGCC…RHD6...ACGAGCCCTTGGTAAGGAAA…PDK<br>Intron...TTGGTCTAGAGATTTCGTCTAGATCGTTTA…RHD6...CCCTACACGACTAGTCCCTAGAGTC…OCS Term	
S8A	qRT-PCR	control gene ACTIN 2	CTCTTAACCCAAAGGCCAAC	ACACACCATCACCAGAATCC
S8A	qRT-PCR	RHD6	CACGAGAGCTTTCCTCCTCC	TTCTGGCCTTGGGGAGTTTC
S8C/D	BSAS	RHD6	GGAAGAGGAGGAATTTTGTTTTGA	CATTCATAACTTCRAARCTTCAAAC

Mutant alleles used in this report

Mutant	Allele	Background Ecotype	Figure
pol V (nrpe1)	SALK_017795	Col	1A/B, 2A/B, 3B, 4A/C/D, 5B/C/D, S1B/D, S2D, S4B/C/D, S7A/D/E, S8B/C/D
suvh2/suvh9	SALK_079574 / SALK_048033	Col	1A/B, 3C/D, 4E
pol IV	SALK_128428	Col	2A/B, S2A/B/C, S7E
drm2	SALK_150863	Col	2A/B
drm1/drm2	CS6366: 6530470652 / 6530470653	WS	1A/B, 2A/B/D/E, 3B, 4D, S1B/D, S7A/D/E
ago4	*ago4-6* (SALK_71772)	Col	2A/B
ago4	*ago4-4* (FLAG_216G02)	WS	2A/B
ago6	*ago6-2* (SALK_031553)	Col	2A/B
ago4/ago6	CS66095: *ago4-1* / *ago6-1*	mixed C24 and Ler	2A
ago4/ago6/ago9	*ago4-4* / *ago6-2* / *ago9-1*	mixed Col and Ws	2A/B/D/E, 3B/C/D, 4D/E, 5C/D, S2D, S5, S7E
dcl1	*dcl1-14* (SALK_056243)	Col	2F/G
dcl2/dcl4	*dcl2-1* (SALK_064627) / *dcl4-2* (GABI_160G05)	Col	2F/G
dcl3	*dcl3-1* (SALK_005512)	Col	2F/G
dcl2/dcl3/dcl4	*dcl2-1* / *dcl3-1* / *dcl4-2*	Col	2F/G
dcl1/dcl2/dcl3/dcl4	*dcl1-9*/ *dcl2-1* / *dcl3-1* / *dcl4-2*	Col	2F/G
cmt2	SALK_012874	Col	S3A/B/C
cmt2/cmt3	*cmt2-7* / *cmt3-11* t	Col	S3A/B/C
met1	*ddm2-1*	Col	S3A/B/C
ddm1	*ddm1-2*	Col	S2D
ago2	SALK_003380	Col	S5
sqk1-1	Crispr promoter deletion allele, from this study	Col	6A/B/C

Supplemental References

1. Gruntman, E. *et al*. Kismeth: Analyzer of plant methylation states through bisulfite sequencing. *BMC Bioinformatics* **9**, 371 (2008).
2. Lanciano, S. *et al*. Sequencing the extrachromosomal circular mobilome reveals retrotransposon activity in plants. *PLoS Genet* **13**, e1006630 (2017).
3. Tsukahara, S. *et al*. Bursts of retrotransposition reproduced in Arabidopsis. *Nature* **461**, 423–426 (2009).
4. Menand, B. *et al*. An Ancient Mechanism Controls the Development of Cells with a Rooting Function in Land Plants. *Science* **316**, 1477–1480 (2007).
5. Sullivan, A. *et al*. An ‘eFP-Seq Browser’ for visualizing and exploring RNA sequencing data. *Plant J* **100**, 641–654 (2019).
