# Supplementary material for: An siRNA-guided ARGONAUTE protein directs RNA polymerase V to initiate DNA methylation
Source: Nat Plants. 2021 Nov 8;7(11):1461–74. doi: 10.1038/s41477-021-01008-7 (PMC8592841; doi:10.1038/s41477-021-01008-7)
Supplement: Supplementary file 2 — Reporting Summary [file 41477_2021_1008_MOESM2_ESM.pdf]

nature research

Corresponding author(s): R.K. Slotkin

Last updated by author(s): 2021-9-2

Reporting Summary

Nature Research wishes to improve the reproducibility of the work that we publish. This form provides structure for consistency and transparency in reporting. For further information on Nature Research policies, see our Editorial Policies and the Editorial Policy Checklist.

Statistics

For all statistical analyses, confirm that the following items are present in the figure legend, table legend, main text, or Methods section.

n/a	Confirmed	
☐	☒	The exact sample size (*n*) for each experimental group/condition, given as a discrete number and unit of measurement
☐	☒	A statement on whether measurements were taken from distinct samples or whether the same sample was measured repeatedly
☐	☒	The statistical test(s) used AND whether they are one- or two-sided<br>*Only common tests should be described solely by name; describe more complex techniques in the Methods section.*
☒	☐	A description of all covariates tested
☒	☐	A description of any assumptions or corrections, such as tests of normality and adjustment for multiple comparisons
☐	☒	A full description of the statistical parameters including central tendency (e.g. means) or other basic estimates (e.g. regression coefficient) AND variation (e.g. standard deviation) or associated estimates of uncertainty (e.g. confidence intervals)
☐	☒	For null hypothesis testing, the test statistic (e.g. *F*, *t*, *r*) with confidence intervals, effect sizes, degrees of freedom and *P* value noted<br>*Give P values as exact values whenever suitable.*
☒	☐	For Bayesian analysis, information on the choice of priors and Markov chain Monte Carlo settings
☒	☐	For hierarchical and complex designs, identification of the appropriate level for tests and full reporting of outcomes
☒	☐	Estimates of effect sizes (e.g. Cohen's *d*, Pearson's *r*), indicating how they were calculated

Our web collection on statistics for biologists contains articles on many of the points above.

Software and code

Policy information about availability of computer code

Data collection	Confirmed - Described in the manuscript.
Data analysis	Confirmed - Described in the manuscript.<br>MethylPy v1.4.6 (https://github.com/yupenghe/methylpy), BedTools v2.27.1, R v4.0.2, Kismeth ((v1.1.0) http://katahdin.mssm.edu//kismeth/revpage.pl), Bowtie v1.2.3, SoapSplice 1.10, ShortStack v3.8.5, Macs2 v2.2.6, GraphPad Prism 9.0.0 were used for processing of data. The custom script used to generate strength of RdDM plots is available on Github - https://github.com/jpeasari/Dot-Plot-Anaysis-OpenCV.

For manuscripts utilizing custom algorithms or software that are central to the research but not yet described in published literature, software must be made available to editors and reviewers. We strongly encourage code deposition in a community repository (e.g. GitHub). See the Nature Research guidelines for submitting code & software for further information.

Data

Policy information about availability of data

All manuscripts must include a data availability statement. This statement should provide the following information, where applicable:

- Accession codes, unique identifiers, or web links for publicly available datasets
- A list of figures that have associated raw data
- A description of any restrictions on data availability

These have been provided in the manuscript. Raw Illumina sequencing data produced for this study is available without restriction from NCBI as GSE165575. Additional small RNA datasets were downloaded from GSE118705. Genome-wide MethylC-seq data is publicly available for wt Col Arabidopsis inflorescence50. Processed data was downloaded from GEO (GSM2101949). ChIP-seq and RIP-seq reads were downloaded from NCBI GEO (GSE52041 and GSE70290). Processed BSAS data of DNA methylation levels is available as Supplemental Dataset 1. Sanger sequencing results are available as Supplemental Dataset 2.

Field-specific reporting

Please select the one below that is the best fit for your research. If you are not sure, read the appropriate sections before making your selection.

☒ Life sciences ☐ Behavioural & social sciences ☐ Ecological, evolutionary & environmental sciences

For a reference copy of the document with all sections, see nature.com/documents/nr-reporting-summary-flat.pdf

Life sciences study design

All studies must disclose on these points even when the disclosure is negative.

Sample size	Sample size depends on which experiment. For qRT-PCR we use 3 biological replicates per genotype in order to be able to test significance. For ChIP, sample size is determined by how many samples were able to be grown and collected and how many samples the researcher can process at the same time. These sample sizes are sufficient for ChIP experiments because they either allow for significance testing or the experiments have been repeated independently. Statistical analysis was not used to determine sample size for any experiment.
Data exclusions	No data was excluded.
Replication	Biological replicates and replicate experiments where used. Entire experimental replication information has been added to the manuscript.
Randomization	Randomization was not applicable to the study.
Blinding	Blinking was not applicable to the study.

Reporting for specific materials, systems and methods

We require information from authors about some types of materials, experimental systems and methods used in many studies. Here, indicate whether each material, system or method listed is relevant to your study. If you are not sure if a list item applies to your research, read the appropriate section before selecting a response.

Materials & experimental systems

n/a	Involved in the study	
☐	☒	Antibodies
☒	☐	Eukaryotic cell lines
☒	☐	Palaeontology and archaeology
☒	☐	Animals and other organisms
☒	☐	Human research participants
☒	☐	Clinical data
☒	☐	Dual use research of concern

Methods

n/a	Involved in the study	
☐	☒	ChIP-seq
☒	☐	Flow cytometry
☒	☐	MRI-based neuroimaging

Antibodies

Antibodies used	Information is provided in the manuscript.<br>Antibodies used include Pol V (custom-made antibody provided as a gift from the lab of Thierry Lagrange, used at 1:1000 dilution), AGO4 (Agrisera cat. no. AS09-617, lot # 1602, used at 1:1000 dilution), ACT11 (Agrisera cat. no. AS10-702, lot # 1105, used at 1:1000 dilution), Pol II Ser5P (Abcam cat. no. ab5131, lot # GR3264297-1), H3K9me2 (Abcam cat. no. ab1220), Rabbit IgG (Cell Signaling Technology cat. no. 2729, lot #10), and Protein A/G magnetic beads (Thermo cat. no. 88802, lot #VK308442)
Validation	The commercially available antibodies have been validated on their respective manufacturers websites. The Pol V antibody was validated in a previous manuscript (Lahmy et al., Genes & Development 2006), as well as in Supplemental Figure 7E using a pol V mutant plant.

ChIP-seq

Data deposition

☒ Confirm that both raw and final processed data have been deposited in a public database such as GEO.

☒ Confirm that you have deposited or provided access to graph files (e.g. BED files) for the called peaks.

Data access links<br>*May remain private before publication.*	All ChIP-seq data in this manuscript are reanalyses of publicly available data. The GEO links and data availability are provided in the manuscript. We will add the BED files for called peaks to the potential revision.

Files in database submission	All the relevant files for the ChIP-seq analysis have already been uploaded to the correct NCBI repository by the authors that originally created that data.
Genome browser session (e.g. UCSC)	N/A

Methodology

Replicates	All ChIP-seq data in this manuscript is reanalysis of publicly available data.
Sequencing depth	All ChIP-seq data in this manuscript is reanalysis of publicly available data.
Antibodies	All ChIP-seq data in this manuscript is reanalysis of publicly available data.
Peak calling parameters	ChIP-seq reads were mapped using ShortStack parameters: --nohp --mmap f --bowtie_m all. The RIP-seq reads were mapped using SoapSplice 1.10 using parameters: -t 10300. Peaks were called with Macs2 using default parameters.
Data quality	We displayed all biological replicates to ensure reproducibility. We also used new negative controls of mitochondrial genes.
Software	MethylPy v1.4.6, BedTools v2.27.1, R v4.0.2, Kismeth, Bowtie v1.2.3, Samtools 1.9, SoapSplice 1.10, ShortStack v3.8.5, FastX toolkit v0.0.13, UEA small RNA workbench v4.5, Macs2 v2.2.6
